# A systematic review and psychometric evaluation of resilience measurement scales for people living with dementia and their carers

**DOI:** 10.1186/s12874-022-01747-x

**Published:** 2022-11-19

**Authors:** Gill Windle, Catherine MacLeod, Katherine Algar-Skaife, Joshua Stott, Claire Waddington, Paul M. Camic, Mary Pat Sullivan, Emilie Brotherhood, Sebastian Crutch

**Affiliations:** 1 Ageing and Dementia @ Bangor, Dementia Services Development Centre (DSDC), School of Medical and Health Sciences, Bangor University, Bangor, UK; 2 Department of Neuromedicine and Movement Science (INB), Norwegian University of Science and Technology (NTNU), Trondheim, Norway; 3 Department of Clinical, Educational and Health Psychology, University College London (UCL), London, UK; 4 4Dementia Research Centre, Queen Square Institute of Neurology, University College London (UCL), London, UK; 5 Faculty of Education and Professional Studies, School of Social Work, Nipissing University, North Bay, ON Canada

**Keywords:** Resilience, Dementia, Carer, Systematic review, Outcome measure, Evaluation

## Abstract

Psychometrically sound resilience outcome measures are essential to establish how health and care services or interventions can enhance the resilience of people living with dementia (PLWD) and their carers. This paper systematically reviews the literature to identify studies that administered a resilience measurement scale with PLWD and/or their carers and examines the psychometric properties of these measures. Electronic abstract databases and the internet were searched, and an international network contacted to identify peer-reviewed journal articles. Two authors independently extracted data. They critically reviewed the measurement properties from the available psychometric data in the studies, using a standardised checklist adapted for purpose. Fifty-one studies were included in the final review, which applied nine different resilience measures, eight developed in other populations and one developed for dementia carers in Thailand. None of the measures were developed for use with people living with dementia. The majority of studies (*N* = 47) focussed on dementia carers, three studies focussed on people living with dementia and one study measured both carers and the person with dementia. All the studies had missing information regarding the psychometric properties of the measures as applied in these two populations. Nineteen studies presented internal consistency data, suggesting seven of the nine measures demonstrate acceptable reliability in these new populations. There was some evidence of construct validity, and twenty-eight studies hypothesised effects a priori (associations with other outcome measure/demographic data/differences in scores between relevant groups) which were partially supported. The other studies were either exploratory or did not specify hypotheses. This limited evidence does not necessarily mean the resilience measure is not suitable, and we encourage future users of resilience measures in these populations to report information to advance knowledge and inform further reviews. All the measures require further psychometric evaluation in both these populations. The conceptual adequacy of the measures as applied in these new populations was questionable. Further research to understand the experience of resilience for people living with dementia and carers could establish the extent current measures -which tend to measure personal strengths -are relevant and comprehensive, or whether further work is required to establish a new resilience outcome measure.

**Supplementary Information:**

The online version contains supplementary material available at 10.1186/s12874-022-01747-x.

## Introduction

Measurement is an essential aspect of scientific research and evaluations of interventions and policies require reliable and valid outcome measures [1]. A twelve-country European working group of researchers and people living with dementia recommend developing new outcome measures that respond to the changing emphasis of dementia research and services towards the possibility of ‘living as well as possible’ with the condition [2], echoing global and national policies [3–5]. The European working group [2] acknowledge the value of constructs such as resilience for outcome measurement to counter the focus on deficit and disease. In response to debates around how to best define resilience, a systematic review and concept analysis of over 270 published articles defines resilience as “*the process of effectively negotiating, adapting to, or managing significant sources of stress or trauma. Assets and resources within the individual, their life and environment facilitate this capacity for adaptation and ‘bouncing back’ in the face of adversity”* [6]. These essential features are corroborated by a recent systematic review of resilience in older people [7]. This ability to ‘do okay’ and achieve good outcomes despite major challenges and stressors is reflected in the growing global interest in healthy ageing [8] and supporting dementia carers [9–11]. Countering the focus on deficit sees emerging research highlighting how people with dementia can ‘live well’ despite the challenges of their dementia [12]. In other words, they are resilient.

Given the interest in resilience, researchers and practitioners may wish to use a resilience outcome measure in evaluation. To ensure data quality, outcome measures require considerable psychometric evaluation, demonstrating they accurately reflect underlying theory and concept, are well-accepted by responders, and accurately measure what they aim to do, in the target population of interest [1]. A number of resilience measures are available for different populations, and their psychometric properties have been systematically reviewed and appraised [13]. Fifteen measures were identified, most (*N* = 10) were developed for application with children and younger populations. None of the measures were developed with, and/or for, people living with dementia or their carer. Most of the resilience measures focus on resilience at the level of the individual only. A strong sense of personal agency may be important for negotiating adversity, but the availability of resources from the wider social environment is also important [13] as captured in recent developments in conceptualising resilience [6].

Another review [14] examined the psychometric properties of six resilience measurement scales in studies which sought to validate the measures in older populations, but none of the studies included people living with dementia or their caregivers. Consequently, it is currently difficult for resilience measures to be considered as one of the set of ‘core outcomes’ which are necessary to reduce the variation and inconsistency in the application of outcome measures in dementia research [15, 16].

Researchers and practitioners are often compelled to make pragmatic decisions regarding the choice of measurement scale, especially as considerable skill and resources are required for developing new outcome measures. Assessors may draw on existing measures originally designed for other populations and use a range of criteria to judge the potential usefulness of the scale, such as previous reports of a scale’s psychometric properties [1]. However, the demographic and circumstantial differences between people living with dementia or their carers, and the population in which the original measure was developed may influence the interpretation, meaning, validity and reliability of the original measure. Psychometric studies are required in order to ascertain whether a measure captures the intended construct (e.g. resilience) in a study sample that may differ from the original scale development sample [17]. The psychometric evaluation of measures is an important area for further investigation if we are to understand how the resilience of people living with dementia and those who support them can be enhanced by health, psychological and social care services or interventions.

A systematic review of positive psychology outcome measures for family caregivers of people living with dementia identified only one study using an existing resilience measure [18]. Stoner and colleagues [19] adapted the wording of the items of a ten-item resilience measure (the Connor-Davidson Resilience Scale), removed two of the items and merged them with an adapted and reduced measure of ‘Hope’ to produce the ‘Positive Psychology Outcome Measure.’ This new measure shows promise for application in populations living with dementia, although the authors did not assess the psychometric properties of the 10-item resilience measure but chose it based on their earlier assessment of the larger 25-item Connor-Davidson Resilience Scale [20]. Consequently, the relevance and appropriateness of existing resilience measures may be inadequate for people living with dementia and their carers and require further investigation.

In response, the present study seeks to contribute new knowledge for research and practice regarding the measurement of resilience. The first aim is to systematically review the literature to identify studies that have administered an established resilience measurement scale with a) people living with dementia and/or b) their carers/supporters (not professional care providers). Identifying existing measurement scales, evaluating their, psychometric properties and possible appropriateness for future adaptation for use in the target population of interest is recommended as a first step in the development of a new measure, should this be required [1]. This leads to our second aim; to examine the psychometric properties of the resilience measures applied in these two populations. We use an established checklist [21] and adapt it to appraise the strengths, weaknesses and usefulness, in order to draw conclusions regarding the measures as applied in these two populations. We discuss the implications of our findings for research and practice.

## Methods

### Search strategy

A systematic review protocol was developed and registered with PROSPERO (https://www.crd.york.ac.uk/prospero/ registration number CRD42021268316). The searches were initially scoped by the lead author and conducted by two people across the Web of Science (provides wide coverage Science Citation, Arts and Humanities Indexes and Social Sciences Citation), PsycINFO, MEDLINE, and ASSIA databases. These were selected so as to enable a comprehensive search beyond the minimum of two databases required to meet critical appraisal standards [22] to source peer-reviewed articles across a wide range of disciplines, e.g. sociology, psychology, health and medicine. Searches were conducted between 22.5.19 and 10.6.19, and updated 5.5.22 using two distinct search arms, combined with the Boolean term ‘AND’ to identify articles. (‘Resilience Or resilient OR resiliency AND Dementia OR Dementia OR Dementia's OR dementias OR demented OR Alzheimer OR Alzheimer's OR Alzheimers OR "posterior cortical atrophy" OR (Benson* AND syndrome) OR "primary progressive aphasia" OR "visual variant"). No date restrictions were applied. To identify further studies, an email was circulated around the INTERDEM^1^ network (May 2019) calling for researchers to contact the lead author if using a measure of resilience. Reference lists of relevant articles were also searched. Experts in resilience recommended further articles as part of the 2022 updated search. All search results were exported into Mendeley and duplicates removed. The original development papers for each of the resilience measures were retrieved by GW.

### Eligibility criteria

**Fig. 1:**
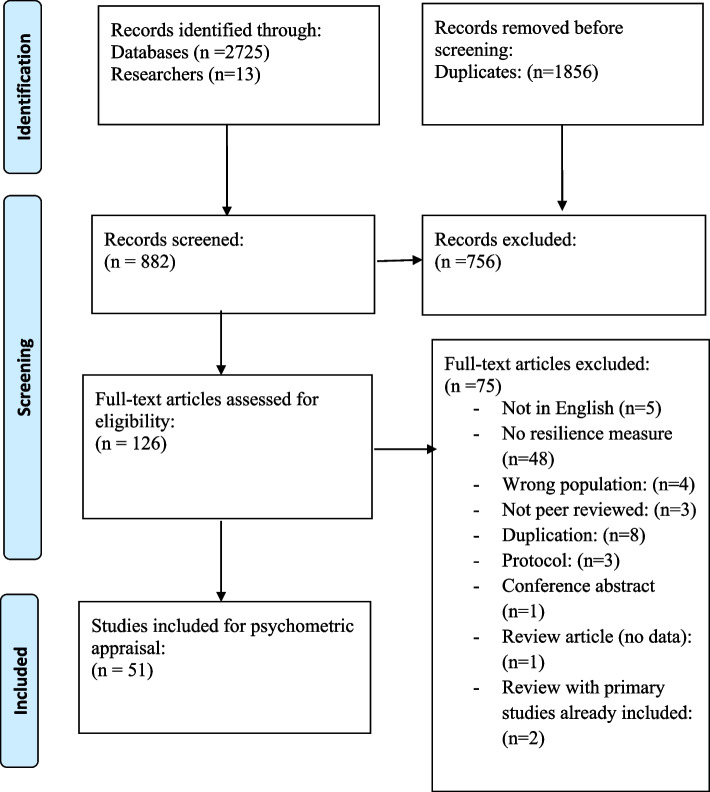
The review process

The titles and abstracts were initially screened by CW and SCu (see acknowledgements). Papers were included if they were an original peer reviewed research/journal article; the sample population was either a person living with dementia or a carer/supporter (family member, friend etc., often described as ‘informal’ carers) and the study used a resilience measurement scale. Papers were excluded if they were an ineligible article type (e.g. conference proceeding, report, book chapter, dissertation); were published in a language other than English; reported qualitative resilience outcomes or were focussed on professional care providers. Disagreements concerning inclusion/exclusion were resolved via a discussion with the lead author. At full-text screening reasons for exclusion were noted. The review process is outlined in Fig. 1.

### Data extraction and analysis

Data were extracted into an EXCEL file, with a worksheet for each of the identified resilience measures. This included information on the study characteristics (sample demographics, purpose of study, country, language, mode of data collection, psychometric data). Psychometric data were appraised using an 18-item checklist with six evaluative domains [21]. This checklist was developed to reflect the main evaluative properties recommended by the Consensus-based Standards for the Selection of Health Measurement Instruments group (COSMIN) [23] and simplified in response to the complexity and length of the COSMIN checklist. See Table 1.

**Table 1:** Scoring criteria for assessment of measurement scales (Adapted from Frances et al. [21])

Checklist Item	Score	Notes
**Domain 1: Conceptual Model** The reasoning for and a description of the concept(s) and the population(s) a measure is intended to evaluate should be specified. Assessments in this domain assists in ascertaining if the measure is likely to capture the intended effect in the population of interest		
1. Construct to be measured has been defined	1 = Yes 0 = No	As per original checklist
2. The intended respondent population has been described	1 = Yes for ‘a’ and ‘b’ and/or ‘c’ 0.5 = Yes for ‘a’ and No for ‘b’ and ‘c’ 0 = No for ‘a’ and/or ‘b’ and ‘c’	Checklist item broken down into 3 parts: a) Study population b) Original measure population c) Authors discuss if measure suitable for their study population
3. Conceptual model addresses whether a single construct / scale or multiple subscales are expected	1 = Yes 0 = No	As per original checklist. Must be explicitly stated
**Domain 2|: Content Validity** The extent to which the questions and sub-scales of a measure are relevant and appropriate for the target population and suitably reflect the concept of interest		
4. There is evidence that members of the intended respondent population were involved in the PRO measure’s development	1 = Yes for ‘a’ 0.5 = No for ‘a’ and Yes for ‘b’ and/or ‘c’ 0 = No for ‘a’ and ‘b’ and ‘c’	Checklist item broken down into 3 parts: a) Related to study b) Related to original measure or previously validated adaptation c) Discuss if original/adaptation involvement is suitable for study population
5. There is evidence that experts in the construct of interest were involved in the PRO measure’s development	1 = Yes for ‘a’ 0.5 = No for ‘a’ and Yes for ‘b’ and/or ‘c’ 0 = No for ‘a’ and ‘b’ and ‘c’	Checklist item broken down into 3 parts: a) Related to study b) Related to original measure or previously validated adaptation c) Discuss if original/adaptation experts are suitable for study population
6. There is a description of the methodology for developing the items/questionnaires (e.g. noting how the respondent population and content experts were accessed and this process generated the questions in the outcome measure)	1 = Yes for ‘a’ 0.5 = No for ‘a’ and Yes for ‘b’ and/or ‘c’ 0 = No for ‘a’ and ‘b’ and ‘c’	Checklist item broken down into 3 parts: a) Related to study b) Related to original measure or previously validated adaptation c) Discuss if original/adaptation methodology is suitable for study population
**Domain 3: Reliability** The level of consistency of an outcome measure, reflected by correlations between the items at a single time point or over time to ascertain whether items or sub-scales are statistically related		
7. There is evidence that the PRO measure’s reliability was tested (e.g. internal consistency, test–retest)	1 = Yes 0 = No	As per original checklist. Must relate to study not original measure or previous studies
8. The reported indices of reliability are adequate and/or justified (e.g. ideal r > = 0.80; adequate r > = 0.70; lower if justified	1 = Yes 0 = No	As per original checklist. Must relate to study not original measure or previous studies
**Domain 4: Construct validity** The extent to which an outcome measure assesses the concept or construct it was designed to reflect		
9. There is reported quantitative justification that single scale or multiple subscales exist in the PRO measure (e.g. factor analysis, item response theory)	1 = Yes 0 = No	Could be related to either current study or original measure
10. There are findings supporting expected (hypothesised) associations with other existing outcome measures or demographic data	1 = Yes for ‘ai’, and ‘b’ and ‘c’ 0.5 = No for ‘ai’ and Yes for ‘aii’, and ‘b’, and ‘c’ OR = Yes for ‘ai’ or ‘aii’, and ‘b’ and ‘c’ for some but not all associations in ‘ai’ and/or hypotheses in ‘aii’ 0 = No for ‘a’ (i or ii) and/or ‘b’ and/or ‘c’	Checklist item broken down into 3 parts: a) i) Known associations between other measures and resilience reported ii) A priori hypotheses of relationship between other measures and resilience b) Results relating to resilience measure reported c) Results match a priori hypotheses and/or known associations (i.e. does ‘a’ match ‘b’)
11. There are findings supporting expected (hypothesised) differences in scores between relevant groups	1 = Yes for ‘ai’, and ‘b’ and ‘c’ 0.5 = No for ‘ai’ and Yes for ‘aii’, and ‘b’, and ‘c’ OR = Yes for ‘ai’ or ‘aii’, and ‘b’ and ‘c’ for some but not all associations in ‘ai’ and/or hypotheses in ‘aii’ 0 = No for ‘a’ (i or ii) and/or ‘b’ and/or ‘c’	Checklist item broken down into 3 parts: a) i) Known differences in resilience by group ii) A priori hypotheses relating to expected differences in resilience by group b) Results relating to resilience measure reported c) Results match a priori hypotheses and/or known associations (i.e. does ‘a’ match ‘b’)
12. The measure is intended/designed to measure change over time	1 = Yes, there is evidence of both test–retest reliability and responsiveness to change OR = There is an explicit statement that the PRO measure is not intended to measure change over time 0 = No	As per original
**Domain 5: Scoring and interpretation** A clear description of how scores on the individual items are calculated to derive the final measure, and an explanation of how differences in scores on a measure are understood		
13. There is documentation how to score the PRO measure	1 = Yes for ‘a’ and ‘b’ 0.5 = Yes for ‘a’ and No for ‘b’ 0 = No for ‘a’	Question broken down into 2 parts: a) Document how measure scored in study b) Measure scored in the same way as originally intended OR discusses why different
14. A plan for managing and/or interpreting missing responses has been described	1 = Yes 0 = No	As per original
15. Information is provided about how to interpret the PRO scores	1 = Yes for ‘a’ and ‘b’ 0.5 = Yes for ‘a’ and No for ‘b’ 0 = No for ‘a’ and ‘b’	Checklist item broken down into 2 parts: a) Information on how to interpret score in study provided b) Interpret score in same way as originally intended OR discusses why different
**Domain 6: Respondent burden and presentation** The time and effort in relation to administering and completing a measure. The literacy level required to complete is suggested to be sixth grade reading level or lower, or the literacy level is adapted for the target population		
16. The time to complete is reported and reasonable?	1 = Yes 0 = No	Where time to complete was not reported, no assessment of the appropriateness of the number of questions was made, as recommended in the original checklist, because of the variability across studies in terms of populations and intended application
17. There is a description of the literacy level of the PRO measure	1 = Yes 0 = No	As per original
18. The entire measure is available for public viewing	1 = Yes 0 = No	As per original

Two authors (GW and KA-S) initially piloted the data extraction, independently reviewing the same two papers. Checklist items regarding content validity relate to the development of the original measure, so the review team agreed to extract any information regarding adaptations to the measures for the population of interest (dementia caregivers and people living with dementia). Two authors (KA-S and CM) then reviewed and extracted data into EXCEL from 51 papers identified during screening meeting the eligibility criteria, regularly discussing and refining assessment criteria throughout the process. This was further reviewed and checked by GW.

The authors of the checklist [21] recommend scoring each item as either ‘0’criterion not met, or ‘1’criterion met.A number of additional data extraction points and a 0.5 score for some items were developed by the current authors to aid the scoring of papers whose focus was not developing a resilience measure, but using existing measures in their research (see Table 1 for checklist, additional points, and scoring adaptations. See Additional File 1 for further description). We include the hypotheses and/or study aims of the included papers to guide the psychometric data extraction. Although not proposed by Francis et al., we established additional evaluative indicators to indicate the strength of the relationship between two measures using Cohen’s criteria [24] where large correlations are > 0.50, medium correlations range between 0.30–0.49 and small correlations range between 0.10–0.29. This additional criteria does not influence the assessment score but is included to facilitate interpretation. Disagreements concerning scoring were resolved via a discussion between GW, KA-S and CM, with the final decision made by the lead author.

We draw on the previous methodological reviews of resilience measures in all populations [13] and resilience measurement in later life [14] to describe the development and psychometric evaluation of the original measures used by the studies in this review. Both the previous reviews addressed the psychometric robustness of resilience measures; Windle and colleagues [13] used a published quality assessment criteria with a scale ranging from 0–18 [25], with the included measures receiving scores ranging from 2–7, concluding that all the measures showed promise but further psychometric evaluation was required, especially in relation to responsiveness. Cosco and colleagues [14] specified the psychometric criteria established from their included studies (e.g. internal consistency, convergent and discriminant validity, construct validity).

## Results and discussion

### Study characteristics

Fifty-one studies were included in the final review [26–76], which applied nine different resilience measures. The review process is documented in Fig. 1. Table 2 describes the characteristics of the included studies and data relating to psychometric properties of the measures (e.g. internal consistency; construct validity; responsiveness). Two studies [42, 66] each report on two different measures so data for each measure is presented separately in Table 2, generating a total of 53 psychometric assessments. The majority of the studies focussed on carers (*n* = 47), three studies focused on people living with dementia, and one study [45] focussed on the dyad (people with dementia and their carers). One study [48] sought to adapt and evaluate the psychometric properties of positive psychology measures (which included a resilience measure, the RS-14) for people living with dementia. Another developed a measure of resilience for carers in Thailand [72]. The remaining studies applied an existing resilience measure (developed in other populations) in their research.

**Table 2:** Characteristics of studies included in the review

Reference (Purpose of study)	Study population Country (language)	Study design (Mode of data collection)	Hypotheses in relation to resilience measure	Relevant psychometric data reported in studies
**Resilience Scale (25 items)**				
Bull [26] (To explore the approaches family caregivers use to help them provide care for a family member with dementia; to describe the psychological distress and resilience of family caregivers)	*N* = 18 family caregivers. 39% were caring for spouses; the others were children of the person with dementia. Age range 37 to 86 years, m = 64 SD = 14.1. 67% were female (67%); 60% were white and 40% were African American. 89% identified themselves as Christian Midwestern USA (English)	Cross sectional mixed-methods design (Telephone interviews)	None specified	None reported
Dias et al. [27] (To investigate the relationship between caregivers’ resilience and the sociodemographic and clinical factors of people with dementia)	*N* = 58 carers (dyads). Most caregivers were female (79.3%), married (77.6%), with a mean age of 62.5 ± 13.4 years. 51.7% were the adult children of the person with dementia Type of dementia: Mild to moderate Alzheimer’s disease, vascular dementia and mixed dementia Rio de Janeiro, Brazil (Portuguese)	Cross-sectional design (Mode of data collection unclear)	Caregivers’ resilience is a personality trait, independent from the clinical symptoms of the person with dementia	Reliability not reported in this study sample No relationship was found between resilience and caregivers’ gender (*p* = 0.883), nor clinical (*p* = 0.807) and emotional problems (*p* = 0.420). There was no significant relationship between caregivers’ resilience and the sociodemographic and clinical characteristics of the person with dementia Large correlations between resilience and caregivers’ depressive symptoms (*r* = -0.539; *p* < 0.01) and carer quality of life (r = 0.514; *p* < 0.01) Medium correlation between resilience and anxiety (*r* = -0.334 *p* < 0.01) Small correlation between resilience and burden (*r* = -0.27, *p* < 0.05). Of these significant univariate associations, depression, and carer QoL significantly predicted resilience. (Effect size for regression not available as authors used SPSS 22)
Fitzpatrick & Vacha Haase [28] (To explore the relationship between resilience and marital satisfaction in caregivers of spouses with dementia)	*N* = 30 caregivers (9 males and 21 females) of spouses with dementia Age range 64 to 90 years, m = 76.4 years, SD = 6.0. One African-American caregiver, the rest were Euro-American Type of dementia: probable Alzeimer’s disease; with 10% of carers reporting MCI, vascular dementia or dementia unspecified Colorado USA (English)	Cross-sectional design (Telephone and face-to-face interviews)	Resilience would be most related to marital satisfaction when adjusting for caregiver burden, age of caregiver, length of marriage in years, gender, and years since dementia diagnosis No hypotheses regarding expected differences in resilience between groups	Ideal internal consistency (α = 0.93) The hypothesis that resilience would be related to marital satisfaction was not supported (*r* = -0.10; probability level not reported) Not hypothesis driven – high correlation between resilience and self-efficacy (*r* = 0.52, *p* < 0.01)
Kimura et al. [29] (To assess whether the clinical symptoms of the person with young onset Alzheimer’s Disease might be associated with resilience in their carers)	*N* = 43 family caregivers; n = 34 were female; (*n* = 21 spouses, *n* = 15 children, *n* = 4 siblings, *n* = 3 ‘other’). 81.4% lived with care recipient. Mean age = 51.1, SD = 15.2 Rio de Janeiro, Brazil (Portuguese)	Cross sectional design (Face to face interviews)	Carer resilience might be related to the presence of awareness of disease, neuropsychiatric symptoms, high levels of depression, and impairment on the functionality of the person with YOAD	Ideal internal consistency (α = 0.80) No significant differences between carers resilience and the characteristics of the person with dementia, contrary to their hypothesis Not hypothesis driven—there are medium negative correlations between carers resilience and their depressive symptoms *(r* = -0.40, p < 0.01); anxiety (*r* = -0.36, p < 0.05), and hopelessness (*r* = -0.33, *p* < 0.05). No significant correlations between carers resilience and carer quality of life
Kimura et al. [30] (To compare the quality of life, burden, and depressive symptoms of caregivers of individuals with young-onset dementia (YOD) and late-onset dementia (LOD))	*N* = 110 dyads of individuals with mild to severe Alzheimer disease and their caregivers (55 dyads of individuals with young-onset Alzheimer disease and 55 dyads of individuals with late-onset Alzheimer disease) *N* = 85 females, mean age = 54.70 (SD = 14.4); *N* = 44 spouses, *N* = 54 children Rio de Janeiro, Brazil (Portuguese)	Cross sectional design (Face to face interviews)	None specified	Reliability not reported for the study sample Not hypothesis driven: YOD caregivers’ resilience: medium correlation with QoL(*r* = -0.365; *p* < .05) and small correlation with depression (*r* = -0.297; *P* < .05) but not burden LOD carers resilience: small correlation with depressive symptoms (*r* = -0.269; *P* < .05) but not burden or QoL
MacCourt, et al. [31] (To report on the structure and effectiveness of a grief management coaching intervention with carers of individuals with dementia)	*N* = 200 Dementia caregivers Spouse (61.9%), parent (23%), other 10%. Mean age = 64.4 (range and *SD* not reported). 79% (*n* = 158) were female; 82% (*n* = 163) were married; 62% (*n* = 122) caring for spouse; 23% (*n* = 65) caring for parent; 5.1% (*n* = 10) caring for ‘other’ Type of dementia: Alzheimer’s Disease or dementia British Columbia, Canada (English)	Controlled mixed methods intervention study (Not reported)	None specified	Ideal internal consistency (time 1 and time 2 α = 0.91) Not hypothesis driven: Responsiveness: There was a significant improvement in resilience from T1 to T2 for the grief coaching intervention group compared to the control group (*F* = 10.70, *df* = 185, p = .009), and there was no change in the control group. Effect sizes not reported
Monteiro et al. [32] (To test the construct validity of the Resilience Scale through exploratory and confirmatory procedures, and to investigate the relationship between caregiver’s resilience and clinical status of people with Alzheimer’s disease)	*N* = 143 carers of people with AD; mean age = 58.8 (SD = 14.3); *N* = 118 females. No other demographic information is presented Rio de Jeneiro, Brazil (Portuguese)	Cross-sectional design (Face to face interviews)	None specified	Adequate to ideal internal consistency α = 0.77 The factor analysis found a four-factor solution—sense of life and self-sufficiency, perseverance, self-confidence and equanimity, and meaningfulness. The authors say this demonstrates the construct validity of the measure, but they do not specify any hypotheses, and note earlier the measure reflects serenity, perseverance, self-confidence, sense of life and self-sufficiency. In view of this, evidence of ‘structural validity’ is difficult to confirm There were no correlations between resilience and PWD clinical measures (functional activities, depression, psychosocial impact, MMSE, NPI, CDR) and between resilience and burden (ZBI)
Pessotti et al. [33] (To ascertain the impact of family caregivers quality of life, burden and resilience and religiosity; to relate these to the clinical and cognitive characteristics of older people with dementia)	*N* = 50 family caregivers. 88% female; mean age = 54.7 (SD = 11.1) 32% were wives and 54% daughters of the person with dementia Type of dementia: 4 people (68%) with Alzheimer’s disease; *n* = 12 with vascular dementia; *n* = 2 with alcohol related; *n* = 2; Parkinson’s Disease related Sao Paulo, Brazil (Portuguese)	Cross-sectional design (Mode of data collection is unclear)	Perceived QoL and burden of carers is more related to aspects of religiosity and more resilient responses, and less associated with clinical aspects of the elder with dementia No hypotheses regarding expected differences in resilience between groups	Reliability not reported in this study sample Results supported the authors hypothesis; high positive correlation between resilience and carers quality of life (*r* = 0.56, *p* < 0.001); medium negative correlations between resilience depression (*r* = -0.36. *P* < 0.01); burden (*r* = -0.36, p < 0.01), and intrinsic religiosity, where lower scores indicate higher religiosity (*r* = -0.37, *P* < 0.01); With the exception of a positive correlation between more severe dementia and higher resilience in a regression model (β = 11.15, *p* < 0.01), there were no associations between caregiver resilience and the socio-demographic and cognitive and disability characteristics of the person living with dementia
Rosa et al. [34] (To investigate the resilience of carers of people with mild and moderate Alzheimer’s, and the related socio-demographic and clinical characteristics)	*N* = 106 caregivers. 72% (*n* = 44) of the *mild AD caregivers* were female and 55.7% (*n* = 34) daughters. 75.4%, (*n* = 46) were married. Mean age = 57.9 ± 13.7 years. 68.9% (*n* = 42) were co-residing with the care recipient. 88.9% (*n* = 40) of *the moderate AD caregivers* women and 48.9%, (*n* = 22) daughters. 71.1% (*n* = 32) were married. Mean age = 59 ± 11.83. 77.7% (*n* = 35) were co-residing with the care recipient Type of dementia: 34 cases (68%) Alzheimer’s disease; *n* = 12 vascular dementia; *n* = 2 with alcohol related; *n* = 2 Parkinson’s disease Rio de Jeniero Brazil (language not reported, but likely Portuguese)	Cross-sectional design (Face to face interviews)	Disease severity may have a direct influence on the resilience of caregivers of PwAD; caregivers of moderate PwAD will have higher levels of burden and lower levels of resilience Co-residing with the PwAD and caregivers’ physical and emotional problems (anxiety and depression) directly contribute to lower levels of resilience among the caregivers of moderate PwAD No hypotheses regarding expected differences in resilience between groups	Reliability not reported in this study sample No differences in the resilience of dementia carers according to whether the person cared for had mild or moderate dementia, contrary to the hypothesis For caregivers of those with moderate dementia, there were small correlations between resilience and the PwAD depressive symptoms (*r* = 0.293; *p* < 0.05), and whether the carer lived with the person with dementia (*r* = 0.299; *p* < 0.05) Medium correlations between carers resilience and the PwAD delusions (*r* = 0.417; *p* < 0.05) and awareness of disease *(r* = -0.374; p < 0.05) and lower levels of carers depressive symptoms (*r* = 0.36; *p* < 0.05) High correlations between higher levels of resilience correlated with carer quality of life (*r* = 0.519; *p* < 0.001) Not hypothesis driven: there were small correlations between the resilience of caregivers of mild dementia and the person’s neuropsychiatric symptoms (*r* = 0.25; *p* < 0.05) and appetite abnormalities (*r* = 0.267; *p* < 0.05) and medium corrleations between resilience and quality of life (*r* = 0.34; *p* < 0.05) Resilience was inversely correlated to the caregivers’ depressive symptoms (*r* =—0.33; *p* < 0.05) and anxiety symptoms (*r* = -0.259; *p* < 0.05)
Svanberg et al. [35] (To explore whether children of younger people with dementia can be compared to other young carers)	N = 12 caregivers (6 male/6 female) Ages ranged from 11 to 17 years (mean 14.6). Eleven were White British and one was mixed race (White-Other) Type of dementia: young onset dementia (*n* = 5 Alzheimer’s disease, *n* = 2 Pick’s disease, *n* = 1 vascular dementia; *n* = 1 suspected Pick’s disease UK (English)	Cross-sectional mixed methods design ( Face to face interviews)	None specified	No relevant data reported
Scott [74] (To explore whether resilience has a moderating effect between Alzheimer’s caregiver stressors and burden)	*N* = 110 caregivers (89 female, 22 male) Ages ranged between 25 and 89 years old (M 63, SD 11) 57 (51.4%) = White, and 52 (46.8%) = Black; 2 (1.8%) = other (self-identified) 40 (36%) = spouse caregivers, 66 (59.5%) = adult–child caregivers 28.8% unemployed, 28.8% full time work, 9% part-time work, 33.3% retired Caregivers provided care for an average of 5 years Type of dementia: Alzheimer’s Disease US (English)	Cross sectional design (Surveys)	Resilience moderates the relationship between identified caregiver stressors and caregiver burden	Reliability not reported in this study sample Contrary to the hypothesis, resilience did not have a moderating effect between caregiver stressors and caregiver burden Not hypothesised: there were no difference in resilience in regards to ethnicity, gender or caregiver type Main effect for resilience (*p* = .001, accounting for approximately 10.2% of the variance in caregiver burden scores. As resilience increased, caregiver burden decreased as demonstrated in Pearson correlation (-.320, p = 0.001) and multiple regression (*b* = -.299, *t* =—4.099, *p* < *.001*) analyses
Garity [73] (To explore the relationship between stress, learning style, resilience and ways of coping in Alzheimer’s caregivers)	*N* = 76 (29% male, 71% female) Mean Age = 61.5 (SD = 14.1) 49% not employed, 31% employed full-time 20% employed part-time 80% married 8% single 7% widowed 5% divorced 43% spouses 33% daughters 9% sons 8% sisters 7% granddaughters 17% 0–1 year care provision, 56% 2–4 years, 19% 5–7 years, 7% more than 7 years Dementia type: Alzheimer’s disease US (English)	Cross sectional design? (Surveys administered in support group)	None specified. Conceptual model of stress used as framework for study	Reliability not reported in this study sample Small correlations were found between resilience and coping style on the emotion-focused subscale of escape avoidance (*r* = -.26, *p* < .05) e.g. less resilience = higher score; and between resilience and the problem-focused sub-scale of planful problem-solving (*r* = 0.30, *p* < .01)
**The Brief Resilience Scale**				
Canevelli et al. [36] (To compare biological age and functional status assessed through the frailty index (FI) in caregivers and matched controls, and (ii) within caregivers, to test the association of FI with measures of perceived psychological stress and resilience)	*N* = 64 caregivers of people with dementia (mean age = 67.62, SD = 11.59; 38 females/26 males) and *N* = 64 non-caregiver controls (mean age = 67.70, SD = 11.63; 38 females/25 males) matched for age and gender Relationship of caregivers: spouses/partners (*n* = 42, 65.6%) or children (*n* = 20, 31.2%) Type of dementia not reported Rome, Italy (Italian)	Cross-sectional matched control design (Mode of data collection unclear)	The authors state: “It can be hypothesized that caregiving, intended as a condition of chronic psychological stress exposure, is associated with accelerated senescence and higher accrual of health deficits, and that, among caregivers, frailty levels are directly related to the intensity of perceived psychological stress and inversely related to the individual’s capacity of psychological resilience, i.e. the capacity of maintaining positive emotional responses in the presence of psychosocial stressors.”	Ideal internal consistency (α = 0.89) Within the caregivers, FI was negatively associated with BRS through a large correlation (*r* = -0.637, *p* < 0.001). This association remained statistically significant (*p* ≤ 0.001) when age, gender, education, BMI, years of caregiving, and type of relationship with the care receiver (i.e. being spouses/partners, children, siblings, or parents of care receivers) were included as covariates Not hypothesised: Resilient caregivers (*n* = 17), i.e. those with high resilience according to the BRS cut-off, had mean FI similar (non-significantly lower) to controls (0.11 ± 0.06 vs 0.16 ± 0.11, F = 2.247; *p* = 0.138). Effect sizes not reported
Chan et al. [38] (1 to explore the caregiver strains and resilience level of caregivers of patients with AD in Malaysia; 2: to determine the factors associated with caregiver strains in caregivers of AD patients, and 3: to determine the effect of resilience on the relationship between caregiver strains and caregivers or patient's factors.)	*N* = 230 carers of people living with Alzheimer’s Disease. 79% (*N* = 165) female. Mean age = 50.4 (SD = 14.5). 59.9% married. Relationship: 16.4% were spouse, 61.4% children, and 17.4% were identified as “others.” Chinese was the majority (57.2%), followed by Malay (13.5%), Indian (8.7%) and 10.6% were Bidayuh, Kadazan and others	Cross-sectional design (Mode of data collection unclear, but possibly self-report/self-completion. Three languages appear to be used-Bahasa Malaysia, English or Mandarin)	No hypotheses specified. The study does not appear to test the theoretical model specified and explores a diverse range of variables. They do not appear to have used the mean scores for the BRS as described in their methods, but used the range	Reliability not reported in this study sample Not hypothesised: The authors applied t-tests and present a range of mean scores for resilience that are not significant according to ethnicity, marital status, education level, kinship, hours of care They note differences in gender with males having higher resilience (M = 20.3, SD = 3.8) than females (M = 18.9, SD = 3.2) *p* = 0.03; and employment status with those unemployed/homemaker/retiree having higher resilience (M = 19.7, SD = 2.9) than those employed full or part time (M = 18.8, SD = 3.5) *p* = 0.04 There were no differences for carers resilience according to levels of functional impairment of the care recipient. There were no differences in carers resilience according to whether or not they lived in the same house, the number of years providing care, whether they received help from family members, emotional support or hired help. Age was not associated with resilience The ‘path’ analysis shows a medium correlation between resilience and caregiver strain (*r* = -0.37, *p* < 0.001)
Kalaitzaki et al. [39] (To identify the perceived symptoms of PwD (i.e. functional impairment, cognitive deterioration and behavioural-psychological symptoms) associated with poorer CGs’ QoL and examine whether CGs’ resilience reduces the effect of dementia symptoms on their QoL)	*N* = 118 caregivers. 78.8% female; mean age = 58.9 (SD = 11.5), 85.6% were married. 90% were the children of the person with dementia Crete, Greece (Greek)	Cross-sectional design (Face to face interview)	None stated	Low internal consistency α = 0.56. The authors suggest this may be due to the brevity of the scale and cite another study suggesting a reliability coefficient of .50–.70 is considered moderately reliable Not hypothesis driven: There was no difference in resilience between those who care few days per week (≤ 3) and those who care many days per week (≥ 4) (18.6 vs. 19.2, respectively; t = .391, *p* = .697) A mediation model found the person with dementia’s behavioural symptoms significantly predicted CGs’ resilience (B = .04, SE = .02, *p* < .05) and CGs’ resilience significantly predicted cares QoL (B = .25, SE = .06, *p* < .001). The direct effect of PwD’s BP on CGs’ QoL (path c) was not statistically significant (B = .02, SE = .01, n.s.), but the indirect effect of PwD’s BP on CGs’ QoL through the mediating role of CGs’ resilience (path c΄) was statistically significant (B = .01, SE = .01, *p* < .05)
McManus et al. [40] (1. To determine the feasibility and acceptability of a performing arts intervention (MPAI) for caregivers of people with mild to moderately severe dementia; 2. To examine how MPAI might change caregiver burden, caregiver resiliency, and perceived quality of life (QoL) for care recipients	N = 32 carers. Age ranged from 41- > 80 mean age not reported. The majority (84%) were female, white (84%) and the spouses of the person with dementia (78%),	Mixed-methods feasibility intervention study with data collected at 5 time-points (Online survey, self completion)	None specified	Internal consistency ranged across the five timepoints from α = 0.79 to α = 0.86 There were no significant differences in resilience scores across the five time-points
Prins et al. [41] (To examine the relationship between involvement of family caregivers (FCs) of people with dementia (PwD) living in LTCFs and FCs mental health during the visitor-ban, and whether this relationship was moderated by the frequency of alternative contact with PwD during the visitor-ban and FC resilience)	*N* = 958 family carers, mean age = 60.30 (SD = 8.95, range between 16 and 89). 71.7% were female. Three quarters (75.7%) indicated that the person with dementia was their father or mother. Spouses or partners represented 10.3% of the sample, while 14.0% had another type of relationship with the person with dementia (for example, other family members, friends, neighbours or legal guardians)	Cross-sectional design (Online survey, self-completion, part of a larger study looking at social isolation during the pandemic)	Hypothesis 1: More family involvement before the visitor banleads to more worries of family caregivers during the visitor ban. This relationship is moderated by the frequency of contact during the visitor ban and the resilience of the family caregiver Hypothesis 2: More family involvement before the visitor ban leads to more experienced loneliness in family caregivers during the visitor ban. This relationship is moderated by the frequency of contact during the visitor ban and the resilience of the family caregiver	Reliability not reported in this study sample. The authors do not use the full scale and reduce it to two items (“I have a hard time making it through stressful events” and “It does not take me long to recover from a stressful event”) No univariate correlations are presented. The role of resilience in hypothesis 1 was not supported The only interaction of resilience was found for carers who undertook task related and social activities before the visitor ban, and also had higher resilience, which predicted lower loneliness (β = -0.32, *p* < 0.05)
Sutter et al. [42] (To examine the relationships between personal strengths (optimism, sense of coherence, resilience) and the mental health of dementia caregivers from Latin America)	*N* = 127 family caregivers ( *n* = 107 from Argentina and *n* = 20 from Mexico).72% (*n* = 98) female; 82% (*n* = 100) married; mean age = 57.14 (SD = 13.01). Relationship: 60% siblings; 22% child; 15.6% spouse; 2.1% partner Type of dementia: Not reported Instituto de Neurociencias de San Lucas,Argentina Baja California, Mexico (Not reported)	Cross-sectional design (Self-report and face to face interview)	None specified	Reliability not reported in this study sample The study reports medium correlations between resilience and satisfaction with life (*r* = 0.32, *p* < 0.01; depression (*r* = -0.35, *p* < 0.01) and large correlations between resilience and optimism (*r* = 0.50, *p* < 0.01). Most of the correlations between resilience and the five RSA subscales were small: personal competence (r = 0.48, *p* < 0.01); social competence (r = 0.23, *p* < 0.01); family coherence (*r* = 0.27, *p* < 0.01); social support (*r* = 0.26, *p* < 0.01); personal structure (*r* = 0.25, *p* < 0.01); and the three Sense of Coherence subscales meaningfulness (*r* = 0.25, *p* < 0.01); manageability (*r* = 0.22, *p* < 0.05) and comprehensibility (*r* = 0.26, *p* < 0.01) In a linear regression (including the RSA subscales and the SoC subscales) resilience (BRS) predicted depression (β = -0.24, *p* < 0.05) but did not predict burden or satisfaction with life. Effect sizes not reported
Vatter et al. [43] (To explore the factor structure of the Zarit Burden Scales (ZBI) in life partners of people with Parkinson related dementia; to examine the relationships among the emerging ZBI factors and demographic and clinical features)	*N* = 136 spouse/life partner caregivers. 85% 9*n* = 116) female; 95% (*n* = 129) married; 89% (*n* = 122) white British; mean age = 69.4 (SD = 7.62) England (English)	Cross sectional design derived from a pilot feasibility study (Face to face interviews and self-completion)	None specified	Reliability not reported in this study sample Not hypothesis driven: The study reports a large negative correlation between carer resilience and the Zarit Burden scale (*r* = -0.53, p < 0.001), and medium and large correlations between carer resilience and five dimensions derived from a factor analysis; social and psychological constraints (*r* = -0.40, *p* < 0.001); personal strain (*r* = -0.50, *p* < 0.001); interference with personal life (*r* = -0.38, *p* < 0.001); concerns about the future *(r* = -0.34, *p* < 0.001) and guilt (*r* = -0.31, *p* < 0.001)
Vatter et al. [44] (To explore and compare levels of mental health, care burden, and relationship satisfaction among caregiving spouses of people with mild cognitive impairment or dementia in Parkinson disease (PD-MCI or PDD) or dementia with Lewy bodies (DLB)	*N* = 136 spouse/life partner caregivers (same participants as reported in Vatter et al., 2018) England (English)	Cross sectional design derived from a pilot feasibility study (Face to face interviews and self-completion)	None specified	Reliability not reported in this study sample Not hypothesis driven: No differences were found for carer resilience across the three types of dementia Medium negative and large correlations between carer resilience and ZBI (*r* = -0.47, p < 0.01); anxiety (*r* = -0.59, p < 0.01); depression (*r* = -0.54, *p* < 0.01); relatives stress (*r* = -0.50, *p* < 0.01); relationship strain (*r* = -0.33, *p* < 0.01); role resentment (*r* = -0.42, *p* < 0.01); role anger *(r* = -0.32, *p* < 0.01). Positive correlations between carer resilience and mental health (*r* = 0.59, *p* < 0.01); health related quality of life (*r* = 0.35, *p* < 0.01); self-rated health (*r* = 0.34, *p* < .01)
Vatter and Leroi [45] (To explore resilience in people with Parkinson’s disease mild cognitive disorder or dementia, or dementia with Lewy bodies, and their care partners, and its relation to outcomes related to their mental well-being and quality of life)	*N* = 76 dyads (*n* = carers and n = 76 people with dementia) Of the participants with Lewy body cognitive disorders, 19.8% (*n* = 15) had a diagnosis of PD-MCI, 52.6% (*n* = 40) had PDD, and 27.6% (*n* = 21) had DLB., 78.9% (*n* = 60) were male, and 93.4% (*n* = 71) were white with a mean age of 74.5 years (SD = 6.74) Of the care partners, 85.6% (*n* = 65) lived with their study partner, 77.6% (*n* = 59) were spouses or partners, 17.1% (*n* = 13) were relatives, and the remainder 5.3% (*n* = 4) included a live-in care partner, a live-in divorcee, a friend, and a grandchild. Of the care partners, 89.5% (*n* = 68) were female, and 92.1% (*n* = 70) were white with a mean age of 65.0 years (SD = 11.81)	Cross-sectional study as part of a pilot feasibility study of an adapted CST for people with Lewy-body related cognitive disorders and their study partners/carers (Face to face interview)	Lower resilience predicts lower mental well-being, quality of life, and relationship satisfaction in both members of the care dyad In care partners, lower resilience predicts higher stress and burden	Reliability not reported in this study sample Carer hypotheses were all met but not all hypotheses for people with dementia were met Care partners self-reported higher resilience scores (m = 3.79, SD = 0.82) than people with Lewy body cognitive disorders (m = 3.23, SD = 0.71, *p* < 0.001) Most participants with Lewy body-related cognitive disorders (74%; *n* = 56) and care partners (83%; *n* = 63) reported high resilience (i.e., above a mean score of 3.00). People with Lewy body-related cognitive disorders with lower levels of resilience had higher levels of anxiety (HADS, *p* < 0.001), higher frequency and severity of neuropsychiatric symptoms (NPI, *p* = 0.047), lower levels of quality of life related to Parkinson’s (PDQ-39, *p* = 0.006), and overall quality of life (EQ-5D, *p* = 0.004) compared to those with higher resilience scores Care partners with lower levels of resilience reported lower relationship satisfaction (RSS, *p* = 0.002), lower quality of life (EQ-5D, *p* = 0.001), lower scores on mental health (SF-12-MCS, *p* < 0.001) and physical health (SF-12-PCS, *p* = 0.037), and higher levels of anxiety (HADS, *p* < 0.001), depression (HADS, *p* < 0.001), burden (ZBI, *p* < 0.001), and stress (Rel.SS, *p* < 0.001) Higher resilience in people with Lewy body-related cognitive disorders was associated with lower anxiety (HADS-A, *r* = -0.52 *p* < 0.001) and higher overall quality of life (EQ5D-VAS, *r* = 0.39, *p* < 0.001) and PD-related quality of life (PDQ-39, *r* = -0.36 *p* = 0.001 – lower scores = better HRQoL) In care partners, medium to high correlations show higher resilience was related to higher relationship satisfaction (RSS, *r* = 0.35,*p* = 0.002), better mental health (SF-12-MCS, *r* = 0.55, *p* < 0.001), and higher quality of life (EQ5D, *r* = 0.38, *p* ≤ 0.002), as well as lower burden (ZBI *r* = -0.44), stress (Rel.SS *r* = -0.44), anxiety (HADS *r* = -0.65), and depression (HADS *r* = -0.54) (all at *p* < 0.001) Multiple regression (note: the methods are unclear as to how this was undertaken). In people with Lewy body-related cognitive disorders, resilience was predicted by anxiety level (F(1,74) = 19.97, *p* < 0.001, adjusted R2 = 0.20), relationship satisfaction (F(1,74) = 4.21, *p* < 0.05, adjusted R2 = 0.04), quality of life (EQ5D-VAS: F(1,74) = 8.51, *p* < 0.01, adjusted R2 = 0.09), and Parkinson’s-related quality of life (PDQ-39: F(1,74) = 11.39, *p* < 0.01, adjusted R2 = 0.12) In care partners, resilience was predicted by: anxiety (F(1,74) = 64.859, *p* < 0.001, adjusted R2 = 0.460), depression (F(1,74) = 31.849, *p* < 0.001, adjusted R2 = 0.291), overall mental health (SF12-MCS: F(1,74) = 31.009, *p* < 0.001, adjusted R2 = 0.286), stress (Rel.SS: F(1,74) = 27.290, *p* < 0.001, adjusted R2 = 0.260), and care burden (ZBI: F(1,74) = 24.749, *p* < 0.001, adjusted R2 = 0.240)
Wuttke-Linnemann et al. [46] (To examine associations among depressive symptoms, partnership quality, and individual resilience in PWD and their caregivers from an intrapersonal, interpersonal, and dyadic perspective and examine the incremental variance explained by interpersonal and dyadic parameters concerning each dyad member’s resilience)	Study 1: *N* = 13 spousal carers, 12 females, mean age = 70.31 (SD = 7.57. *N* = 13 people with dementia, 12 males, mean age = 75.85, (SD = 4.69) Study 2 N = 16 spousal carers, 11 females, mean age = 74.75 (SD = 6.79). *N* = 16 people with dementia, 5 females, mean age = 76.94, (SD = 6.75) Type of dementia: Alzheimer’s Disease Germany (German)	Cross-sectional secondary analysis of data from two intervention studies	H1: The prediction of each person’s resilience score can be incrementally increased by adding the scores of the respective partner H2: Dyadic interdependencies among the dyad in self-rated depression and self-rated partnership quality predict individual perceptions of resilience	Reliability not reported in this sample Carers: there were no significant correlations between resilience, depression and the marital quality questionnaire People with dementia: medium and large correlations between resilience and depression (*r* = -0.44, *p* < 0.05) and marital quality (r = 0.52, *p* < 0.01) H1: not supported in the carers or the person with dementia – their individual resilience was not enhanced by their spouses data H2: The authors indicate the interdependencies were calculated by creating a ‘similarity score’ by calculating the negative squared difference between two measures. These were then standardised as Z scores. They report a similarity in depression scores is associated with lower individual resilience in caregivers and with higher individual resilience in patients
**Resilience Scale 14 (RS-14)**				
D’Onofrio et al. [47] (To illustrate the key results and evidence obtained in the final evaluation of the Mario robot)	*N* = 38 people living with Alzheimer’s Disease (M = 14; F = 24). Age range 55–93 years (m = 77.08 ± 9.91 years). Ethnicity not reported Galway, Ireland (English) Rotondo, Italy (Italian) Stockport, England (English)	Feasibility study with pre-post evaluation of a robot intervention for people living with Alzheimer’s Disease (Face to face interview)	None specified	Reliability not reported in this study sample No correlations reported between resilience and other measures Not hypothesised: The MARIO robot significantly increased resilience scores between pre (*M* = 31.33, *SD* = 21.45) and post (*M* = 36.96, *SD* = 15.35) intervention (*p* = 0.02)
McGee et al. [48] (To adapt and evaluate the psychometric properties of positive psychology measures for people living with dementia)	*N* = 36 people living with early-stage dementia (most frequent diagnosis being Alzheimer’s Disease). Age range 56 to 93 (M = 74.39,SD = 10.70). 61% were female and 67% were married. 33% (*n* = 12) were educated to Bachelor level or above Southern USA (English)	Cross sectional design (Self-completion)	The authors do not define specific hypotheses, but state they test the convergent validity of their positive psychology measures (resilience, optimism, meaning in life, gratitude, life satisfaction) and discriminant validity between the positive psychology measures, depression and anxiety (p.311)	Ideal internal consistency (α = 0.81) Medium correlations between resilience and the ‘presence’ subscale of the meaning in life (*r* = 0.48, *p* < 0.01); optimism *(r* = 0.38, *p* < 0.05); and gratitude (*r* = 0.39, *p* < 0.05), and large correlations between resilience and depression *r* = -0.54, *p* < 0.01) and anxiety (*r* = -0.72, *p* < 0.01) Resilience did not correlate with life satisfaction or the ‘search’ sub-scale of the meaning in life scale
Orgeta et al. [49] (To evaluate the clinical effectiveness and cost-effectiveness of carer-delivered individual cognitive stimulation therapy for people with dementia and their family carers, compared with treatment as usual)	*N* = 356 caregivers. 73% (*n* = 261) female; 92% (*n* = 321) white ethnicity; 66% (*n* = 236) living with spouse/partner with dementia; mean age = 65.73 (SD = 12.92) Type of dementia: 64% (*n* = 227) Alzheimer’s Disease; 11% (*n* = 40) vascular dementia; 3% (*n* = 11) Lewy Body; 12% (*n* = 41) unknown England and Wales, UK (English)	A multicentre, single-blind, randomised controlled trial assessing clinical effectiveness and cost-effectiveness. Assessments were at baseline, 13 weeks and 26 weeks (primary end point) ( Face to face interview)	iCST will improve the primary and secondary outcomes (including resilience) compared to TAU	Reliability not reported in this study sample There were no significant differences in resilience over time between carers accessing iCST and carers receiving treatment as usual
Sánchez-Teruel et al. [50] (To identify the variables that predict a high degree of well-being in family caregivers of people with dementia during the Covid-19 lockdown)	*N* = 320 carers; *N* = 266 females; age range 20–73, mean age = 46.45, SD = 15.97). *N* = 205 were educated to degree level/vocational training or higher Type of dementia as described by authors: AD = 82, ‘senile’ = 88, Parkinson = 67, vascular = 63, other = 10 Spain (Spanish)	Cross-sectional design (Self-completion/online survey)	None stated	Ideal internal consistency (α = 0.88) Not hypothesised: Large correlations between resilience and well-being (*r* = 0.92; *p* < 0.01), self-efficacy (*r* = 0.78; *p* < 0.01), coping strategies (*r* = 0.65, *p* < 0.01), self-compassion (*r* = 0.59, *p* < 0.01) and difficulties in emotion regulation (*r* = -0.88, *P* < 0.01) Multiple regression found resilience predicted well being along with other variables but the lack of hypotheses make the interpretation difficult
Stansfeld et al. [51] (To undertake a psychometric evaluation of the Sense of Coherence Scale)	*N* = 583 caregivers. 80.3% were female Age range between 18–89, M = 59.5 years SD = 12.3. 94% were white British or Irish. 69% were married, 59% were the son or daughter and 30% the spouse Type of dementia: Alzheimer’s disease (50.5%), vascular (18.9%), DLB (3.3%), FTD (23.3%) UK (English)	Cross-sectional mixed methods design ( Self-completion)	Sense of coherence will be positively correlated with caregiver’s resilience No hypotheses regarding expected differences in resilience between groups	Reliability not reported in this study sample As hypothesised, carers resilience positively correlated with sense of coherence (*r* = 0.56, *p* < 0.001) Not hypothesised: small correlation between resilience and sense of competence (*r* = 0.25, *p* < 0.001); medium correlation between resilience and self-efficacy (*r* = 0.45, *p* < 0.001) and high correlation between resilience and health related quality of life (*r* = 0.56, *p* < 0.001)
Wilks et al. [52] (To explore the moderating effects of spiritual support on the relationship between caregiver burden and resilience)	*N* = 684 caregivers, 80% were female. Mean age = 61, range not reported. 62% (*n* = 426) were married. 62% (*n* = 424) reported their ethnicity as Caucasian 36% (*n* = 246) reported as African American. 51% (*n* = 350) were the child of the care recipient	Cross sectional secondary data analysis (Self-report)	There will be no significant ethnic difference in the relationship between care giving burden and the resilience outcome There will be no significant ethnic difference regarding the relationship between spiritual support and the resilience outcome Spiritual support will not serve as a significant moderating factor among the risk- resilience relationship among African American caregivers. A similar, non significant result for Caucasian caregivers will be found	Reliability not reported in this study sample Spiritual support significantly predicted resilience in African American carers (β = 0.36, *p* < 0.01) and Caucasian carers (β = 0.01, *p* < 0.05) Caregiver burden did not predict resilience in either ethnic group [The methods for the analyses in this paper are unclear]
Wilks et al. [75] (To examine whether caregiver coping strategy moderates the relationship between aggression in Alzheimer’s and caregiver resilience; whether aggression is associated with specific, caregiver coping strategies; whether aggression is associated with diminished caregiver resilience)	*N* = 419 (330 female, 86 male) Mean age = 61 Caucasian/White = 57%, *n* = 236, African American/Black = 41%, *n* = 171children of care recipients = 52%, *n* = 215, spouse/partners = 15%, *n* = 64 Stage of AD = early (*n* = 72, 18.4%), middle (*n* = 156, 39.9%), late (*n* = 161, 41.2%) Dementia type: Alzheimer’s disease US (English)	Cross sectional design (data obtained via self-report and Likert scales)	None specified	Ideal internal consistency (α = .94) All strategies of coping correlated significantly with RS14 scores. Specifically, RS-14 scores positively correlated with CITS-task (*r* = 0.39, *p* < 0.01) and negatively correlated with CITS-emotion (*r* = -0.19, *p* < 0.01), CITS-avoidance (*r* = -0.16, *p* < 0.01) and RMPBC aggression (*r* = -0.11, *p* < 0.05) scores Reported significant interaction of RMBPC aggression × CITS-emotion (β = − .15). and RMPBC aggression × CITS-avoidance (β = − .12) on predicting RS14 scores Instead of moderation, the negative effect of RMBPC aggression (β = − .11) increased upon the interaction of CITS-emotion and CITS-avoidance, respectively No interaction of RMBPC aggression × CITS-task on predicting RS14 scores (β = .01) Task-focused coping accounted for the most variability in RS-14 scores (23%) which more than doubled the effect sizes of emotion-focused (11%) and avoidance-focused (10%) strategies
**Connor-Davidson Resilience Scale (CD-RISC)**				
Duran-Gomez et al. [53] To assess the resilience of caregivers of people with AD	*N* = 140 carers; 104/86.7% female; mean age = 50.5, SD = 4.2. 74% married, 68 lived with the person they provided care for Badajoz, Spain (Spannish)	Cross sectional design (Face to face interview)	High scores in resilience will be associated with exposure to a lower number of stressors derived from care, perceived stressors caregiver assessment, and adequate physical and psychological state of health and better quality of life Resilience will be related to other mediator variables, such as perceived social support and the intrapsychic resources of the caregiver (coping styles, self-esteem or sense of competence)	Reliability not reported in this study sample Carers mean score = 69.24 ± 14.07. A cut-off score of 70 identify highly resilient caregivers (= / > 51.66% of the sample) Medium correlations between resilience and lower levels of dependency (*r* = − 0.417, *p* < 0.01), care recipient’s cognitive decline (*r* = − 0.393, *p* < 0.01) and large between resilience and carer burden (*r* = − 0.623, *p* < 0.01), but not with NPI scores Resilience correlated with anxiety (*r* = -0.33, *p* < 0.01); depression (*r* = -0.51, *p* < 0.01), HRQoL (*r* = 0.58, *p* < 0.01); role-physical (*r* = -0.19, *p* < 0.05) but not any other of the SF-36 domains Resilience correlated with self esteem (*r* = 0.04, *p* < 0.01); social support (*r* = 0.22, *p* < 0.05); emotional support (*r* = 0.31, *p* < 0.01); positive social interaction (*r* = 0.20 *p* < 0.02), problem focussed coping (*r* = 0.36, *p* < 0.01) but not emotion focussed or avoidance focussed coping. All significant variables were entered in a linear regression, and burden, anxiety, coping, social support, cohabitation, help provided and HRQoL all predicted resilience. Effect sizes not reported for regression
Gómez-Trinidad et al. [54] (To analyze the relationship of resilience and emotional intelligence with functional performance in the main caregivers of people with dementia in Spain according to the severity of the disease)	*N* = 144 carers; 70% female, 79% married, mean age not presented 67% of the care recipients had Alzheimer’s Disease, with 61.1%(88) in the moderate stages and 25.7%(37) severe Spain (Spanish)	Cross-sectional design (Self-completion)	None stated	Reliability data not reported in this study sample Not hypothesised; Small correlations between resilience and longer time spent on self-care (*r* = 0.196; *p* = 0.019) and leisure (r = 0.172; *p* = 0.040). The time dedicated to productivity was not related to the level of resilience (*r* = 0.091; *p* = 0.278). These variables did not correlate when looking specifically at carers of people with mild phase dementia; for carers of people in the moderate phase: small correlations between resilience and the time dedicated to self-care (*r* = 0.227; *p* = 0.033) and leisure (*r* = 0.262; *p* = 0.014). For carers of people with severe dementia, a medium correlation between resilience and the time dedicated to productivity (*r* = 0.355; *p* = 0.034)
Lavretsky et al. [55] (A double-blinded placebo-controlled trial to investigate the efficacy of an antidepressant drug, Escitalopram, to improve depression, resilience to stress, and quality of life in depressed family dementia caregivers)	*N* = 40 caregivers (age range 45–91, 25 adult children and 15 spouses; 26 women) Type of dementia: Alzheimer’s Disease California, USA (English)	A double-blinded placebo-controlled trial (Mode of data collection unclear)	Escitalopram would improve resilience in those receiving the drug compared to those in the control arm	Reliability data not reported in this study sample The authors note participants who took Escitalopram showed an improvement in resilience, but the data is not presented/unclear
Rivera-Navarro et al. [56] (To validate the Caregiver Abuse Screen (CASE) as an instrument for detecting the maltreatment of people with dementia in Spain)	*N* = 326 carers. Most were women (67.2%) and offspring or spouses (93.9%), mean age = 60.1 years (SD = 14.5) Northwest Spain (Spanish)	Cross sectional design (Face to face interview)	The CASE may be a reliable instrument to measure different components of maltreatment (i.e., interpersonal abuse and neglect) in Spain A positive and statistically significant relationship between risk factors of maltreatment (e.g., burden, anxiety and depression in caregivers, functional dependence and cognitive impairment in people with dementia) and CASE scores, will be found Caregiver resilience and caregiver social support, as protective factors, will show a negative association	Reliability data not reported in the study sample As hypothesised, medium correlations were found between resilience and the CASE (*r* = -0.350, *p* < 0.01) and the CASE subscales interpersonal abuse (*r* = -0.30 *p* < 0.01) and a small correlation with neglect/dependency (*r* = -0.28, *p* < 0.01)
Ruisoto et al. [57] (To examine different predictive factors of burden in a sample of family caregivers of patients with dementia (PWD))	*N* = 283 carers. *N* = 186 females; mean age = 59.93 (SD = 65.72). *N* = 157 were children/children in law and N = 115 were spouses Spain (Spanish)	Cross sectional design (Face to face interview)	None stated	Reliability not reported in this study sample Not hypothesised: a small negative correlation between resilience and burden (*r* = − 0.218, *p* < .001) and resilience predicted burden in a regression model. The authors test a model where social support mediates the relationship between resilience and burden, but it isn’t clear why this model was tested
Serra et al. [58] (To understand the associations between abuse related behaviour and key characteristics of dementia carers and the person they care for)	*N* = 283 caregivers who lived with the person with dementia (Son/daughter = 115/40.6; husband/wife = 157/55.5%)67% were female. Mean age = 59.9 ± 14.6 Type of dementia: Alzheimer’s Disease (85%); ‘other’ 14.5% Castilla and León, Northwest Spain (Spanish)	Cross-sectional design (Some data collected through an interview, but unclear how the resilience scale was administered)	Resilience is expected to decrease the possibility of abuse No hypotheses regarding expected differences in resilience between groups	Reliability data not reported in the study sample Abuse scores measured the CASE correlated negatively with resilience (*r* = − .35, *P* < .001), and in a linear regression, carer resilience predicted lower levels of abuse (β = -0.28, *p* < 0.001)
Wilks & Vonk [76] (To determine whether private prayer acts as a mediator for caregiver burden and perception of resiliency in Alzheimer’s caregivers)	*N* = 304 (233 female) Mean age = 63 (SD = 13.5) 86% White (n = 261), 13% African American (*n* = 40), 1% Hispanic (*n* = 2) married (79%, *n* = 240),10% divorced (*n* = 31), 5% single (*n* = 14), 5% widowed (*n* = 14 spouses (43%, n = 131), 39% children(*n* = 118), 4% friends (*n* = 13), 14% “other” (*n* = 42) Type of dementia: Alzheimer’s Disease US (English)	Cross sectional design (Self report: Questionnaires)	None specified	Reliability not reported in the study sample Participants reported a moderate-to-high level of perceived resiliency. Burden correlated negatively with perceived resiliency (*r* = -.53, *p* < .01), with higher burden associated with lower resiliency. Burden accounted for approx. 16% of the variation in perceived resiliency. Further, as prayer coping increased, so did perceived resiliency (*r* = 0.23, *p* ≤ 0.05) With the inclusion of private prayer, the indirect effect of caregiving burden on perceived resiliency decreased (-.49), with private prayer accounting for 7% of variation in perceived resiliency Burden and private prayer accounted for 23% of the variation in perceived resiliency
**Connor Davidson Resilience Scale 10 (CD-RISC 10)**				
Bravo-Benitez et al. [59] (The objectives were to adapt a grief intervention program to family caregivers of patients with dementia and assess its effectiveness in improving their symptoms of grief and other health-related variables)	*N* = 52 family caregivers Mean age = 63.88 years (SD = 17.55; range: 21–89), 21.15% were male and 78.85% were female. Relationship: 57.69% spouses, 34.62% children, and 7.69% were other relatives. 7.69% no education, 26.92% had primary education, 19.23% had secondary education, and 46.15% had higher education Grenada, Spain (Spanish)	A repeated measures quasi-experimental randomized design with allocation of participants to either the intervention group (IG) or to the control group (CG) (on a waiting list). Randomisation process not described (Interviews undertaken at the Association of Relatives of patients with AD Centre in Grenada)	No hypotheses stated in relation to the resilience measure The authors state “It was expected that caregivers who participated in this intervention program would exhibit significant improvements in their overall perceived health, quality of life, as well as a significant decrease in maladaptive manifestations associated with grief.”	Reliability not reported in this study sample (the authors cite the original development paper) Not hypothesised: significant differences were found in the Time × Group interaction [F(1, 50) = 16.961; *p* < 0.001]. A decrease in resilience was observed in the CG between the pre (M = 29.60) and post (M = 26.04) assessment, and increased in the IG between pre (M = 23.74) and post (M = 27.89) assessment. The groups are not balanced at baseline and there is no main effect of time
Carbone et al. [37] (1: To explore changes due to the COVID-19 lockdown in the BPSD of community-dwelling PwD and the distress experienced by their family caregivers; 2: to explore the associations between caregivers’ ratings of the frequency and severity of their relative’s BPSD and of their own related distress; 3: to explore caregivers’ perceived social and emotional loneliness, and resilience, i.e., the ability to cope with adversity, and adapt to the physical and psychological challenges of caregiving)	*N* = 35 family caregivers of community dwelling people with dementia. Relationship: *N* = 34 were family members (spouses, children, or siblings), while one was a paid living-in carer. *N* = 26 female (74.28%) Type of dementia: Alzheimer’s disease 17.1%; vascular dementia 37.1%, and mixed or other types of dementia 60% Trevisio, Italy (Italian)	Cross-sectional design (Telephone interview)	The authors state: “We expected a greater degree of resilience to be associated with lower caregiver ratings, and fewer reported lockdown-related changes in the frequency and severity of the BPSD in their RwD, and in their own related distress.”	Reliability not reported in this study sample Not all hypothesised relationships confirmed. The authors found a medium correlation between resilience and changes in total NPI caregiver distress scores (*r* = -0.32, *p* < 0.01). The authors suggest this indicates that greater resilience was associated with a more limited worsening under lockdown of the distress experienced by caregivers regarding the BPSD of their RwD Not hypothesised: female gender was associated (medium) with higher resilience scores (*r* = 0.32, *p* < 0.05)
Sarabia-Cobo and Sarria [60] (To examine Sense of Coherence, Resilience and Emotional Regulation as predictors of satisfaction with care in caregivers of older adults people with dementia)	*N* = 63 caregivers; 85.7% were women (*n* = 54), 82.5% were unemployed (*n* = 52). The mean age was 63.40 (SD: 14.92)	Cross-sectional design (Telephone or Zoom interview)	Higher levels of Sense of Coherence, Resilience and Emotional Regulation in caregivers will be associated with greater satisfaction with caregiving	Adequate to ideal internal consistency (α = 0.77) Satisfaction with care was negatively associated with Resilience (*r* = − 0.65, *p* < .001). The authors state the negative correlation is due to the lower values of the satisfaction scale which relates to greater satisfaction (higher scores = lower satisfaction) Resilience was associated with Sense of Coherence (*r* = 0.97, *P* < 0.05); and Emotional Regulation (*r* = 0.25, *p* < 0.05)
**Dispositional Resilience Scale**				
O’Rourke et al. [61] (To determine if three aspects of psychological resilience (commitment, control, challenge) of dementia carers predicts lower levels of depression one year later; to ascertain if change in resilience occurs simultaneously with change in depression between measurement points)	*N* = 105 cohabiting spouses (*n* = 58 wives/*n* = 47 husbands). Mean age = 69.59 years (SD = 8.66; range 46–89). M = 14.36 years of formal education (SD = 3.27) Type of dementia: Probable or possible Alzheimer’s disease Vancouver, Canada (English)	Longitudinal cohort design (Mode of data collection unclear)	Psychological resilience would (inversely) predict depressive symptoms one year later; in addition, a reported increase in resilience between points of measurement would correspond to a further reduction in depressive symptoms over this interval No hypotheses regarding expected differences in resilience between groups	Reliability not reported in this study sample The authors hypotheses were partially supported. Higher control scores at baseline predicted lower levels of depression a year later (ƴ = -1.17, SE = 0.28, *p* < 0.005). Higher challenge scores at baseline predicted lower levels of depression a year later (ƴ = -1.77, SE = 0.46, *p* < 0.005). The increase in the challenge score between the two time points predicted a reduction in depressive symptoms over time ( ƴ = -7.74, SE = 3.38, *p* < 0.05). No significant findings for commitment as a predictor of depression at time 2, or as a predictor of change in depression scores over time
**Resilience Scale for Adults (RSA)**				
Altieri & Santangelo [62] (To explore changes in depression and anxiety in caregivers of people with dementia during the Italian Covid-19 lockdown, and the extent to which these differed by low and high resilience)	*N* = 84 caregivers (71 females; 13 males). Relationship: 72.6% children, 11.9% spouses, 8.3% grandchildren, 6% other. 75% lived in same house. Mean age 48.7 years (SD = 11.7). Type of dementia 56% AD, 31% VD, 10.7% FtD, 2.4% LBD Italy (Italian)	Cross-sectional design (Online survey)	None stated	Reliability not reported in this study sample Repeated measures ANOVA found a significant: 1) main effect of resilience level between subjects (L = 0.795, F(2,81) = 10.445; *p* < 0.001, partial h2 = 0.969), 2) main effect of time (L = 0.865, F (2,81) = 6.297, *p* = 0.003, partial h2 = 0.135), and 3) interaction between time and resilience (L = 0.910, F (2,81) = 4.013, *p* = 0.022, partial h2 = 0.090) Univariate analysis found and a significant interaction between time and resilience on HADS-A scores (F(1,82) = 6.955, *p* = 0.010, partial h2 = 0.078) but not on HADS-D scores (F (1,82) = 2.987, *p* = 0.088, partial h2 = 0.035). There was a significant between subjects effect of resilience levels on HADS-D (F(1,82) = 19.644, *p* < 0.001, partial h2 = 0.193) and HADS-A (F(1,82) = 7.811, *p* = 0.006, partial h2 = 0.087) scores Higher Caregiver Burden Inventory scores were negatively associated with RSA scores (b = -.398, t(81) = 3.644, *p* = 0.001)
Elnasseh et al. [63] (To examine whether healthier family dynamics were associated with higher personal strengths of resilience, sense of coherence, and optimism among dementia caregivers in Argentina)	*N* = 105 family caregivers (relationship not reported, status is ‘primary’ caregiver). 74% were female, mean age 57.71 years (SD = 13.35). 75% female, 32% completed college Type of dementia not reported Rosaria, Argentina (Spanish)	Cross-sectional design (Face to face interviews)	Healthier family dynamics will be related to a higher sense of coherence, greater resilience, and more optimism Greater family communication is hypothesized to be asssociated with resilience Empathy is hypothesized to be associated with resilience No hypotheses regarding expected differences in resilience between groups	Ideal internal consistency (α = 0.96). The authors also note that ‘Adequate reliability has been shown for each subscale with α’s ranging from 0.67 to 0.90’. It is not clear if this relates to the current study, but in the absence of a reference to another study, we assumed it relates to the current study Medium correlations between caregiver resilience and empathy (*r* = 0.33, *p* < 0.01); cohesion (*r* = 0.38, *p* < 0.01); communication (*r* = 0.40, *p* < 0.01); family satisfaction (*r* = 0.42 *p* < 0.01) and sense of coherence (*r* = 0.423 *p* < 0.01), and a small correlation between resilience and supporting the hypotheses flexibility (*r* = 0.22, *p* < 0.05); Caregiver resilience also correlated with income (*r* = 0.33, *p* < 0.001)
Gulin et al. [64] Aim: To examine the influence of resilience, coping and optimism on the quality of care provided by dementia carers in Argentina and Mexico	*N* = 110 caregivers from Argentina and *N* = 20 from Mexico. Mean age = 56.84 years (SD = 13.18). 77.7% female. Relationship: 43.8% spouses, 43.1% children, 7.7% uncles/aunts, 2.3% ‘other’, 1.5% professional caregivers, 0.8% friends, 0.8% parents Type of dementia: Not reported Rosario, Argentina and Baja California, Mexico (Spanish)	Cross-sectional design (Face to face interviews)	Greater personal strengths will be associated with higher quality of informal care No hypotheses regarding expected differences in resilience between groups	Ideal internal consistency (α = 0.95). The analysis does not use the full scale but examines each of the 5 sub-scales and no reliability statistics are presented for the sub-scales Of the five resilience sub-scales, family coherence predicted quality care (β = 0.33, SE = 0.04, *p* < 0.001) and respectful quality of care (β = 0.34, SE = 0.06, *p* < 0.001), suggesting minimal support for their hypothesis
Senturk et al. [65] (To investigate the relationship between caregiver burden and psychological resilience of caregivers individuals with dementia)	*N* = 103 caregivers. The mean age of caregivers is 56.5 ± 9.91 85.4% of them are female, 42.7% of them have an undergraduate degree, 42.7% of them provide care for their mother, Turkey (language not stated)	Cross-sectional design (Mode of data collection not stated)	None specified	Ideal internal consistency (α = 0.82) There was a large negative correlation between caregiver burden and psychological resilience (*r* = -0.869, *p* < 0.001)
Pandya [66] (To examine the impact of a long-term meditation program in enhancing self-efficacy and resilience of home-based caregivers of older adults with Alzheimer’s in two South Asian Cities)	185 carers at the pre-test phase (96 in the intervention group and 89 in the control group) and 145 participants at the post-test phase (78 intervention group and 67 control group) Mean age pre test control group 52.5(10.67) intervention group 52.68(11.03), post test control group 57.6(11.32) intervention group 58.02(10.38) Females pre test control group *N* = 77/86.52%; pre-test intervention group N = 78/81.25%; post test control group *N* = 59/88.06%; *N* = 67/85.90% Male 12 13.48 18 18.75 8 11.94 11 14.10 Female 77 86.52 78 81.25 59 88.06 67 85.90 Mumbai, India and Kathmandu, Nepal (English)	Intervention study with post-intervention data collected five years later (Mode of data collection unclear)	Hypothesis 1: Meditation program would reduce the perceived caregiving burden overload and enhance self-efficacy and resilience of home-based caregivers of older adults with Alzheimer’s. Hypothesis 2: It is hypothesized that the intervention group participants’ characteristics as well as program-related characteristics would predict different levels of self-efficacy and burden. Hypothesis 3: Home practice by caregivers would be the strongest predictor of perceived caregiving burden reduction, caregiving self-efficacy enhancement and increased resilience	Cronbach’s α = .92; item-scale intercorrelation = .89; P The authors state there was no significant difference between intervention and control groups on socio-demographic and caregiving characteristics Post-test RSA scores were higher in the intervention than the control group (mean difference = 87.92, *p* = .001, d = 7.55) and their own pre-test scores (mean difference = 87.62, *p* = .001, d = 7.36). The authors state “Post-hoc analyses indicated that the post-test perceived caregiver burden overload scores were lower and self-efficacy and resilience scores were higher for women caregivers, caregivers who were spouses of older adult patients, Hindus, middle class, with college and higher education, homemakers, who attended at least 187 (i.e. at least 75%) meditation lessons and regularly practiced at home (i.e. once weekly for 187 weeks or at least 75% weeks)
Sutter et al. [42] Aim: To examine the relationships between personal strengths (optimism, sense of coherence, resilience) and the mental health of dementia caregivers from Latin America	*N* = 127 family caregivers (*n* = 107 from Argentina and *n* = 20 from Mexico).72% (*n* = 98) female; 82% (*n* = 100) married; mean age = 57.14 (SD = 13.01). Relationship: 60% siblings; 22% child; 15.6% spouse; 2.1% partner Type of dementia: Not reported Instituto de Neurociencias de San Lucas,Argentina Baja California, Mexico (Not reported)	Cross-sectional design (Self-report and face to face interview)	None specified	Reliability not reported in this study sample The authors do not present data for the total score of the RSA, but the five sub-scales. They also included another measure of resilience (the BRS) the Brief Resilience Scale correlated with the five RSA sub-scales: personal competence (*r* = 0.48, *p* < 0.01); and small correlations with social competence (*r* = 0.23, *p* < 0.01); family coherence (*r* = 0.27, *p* < 0.01); social support (*r* = 0.26, *p* < 0.01); personal structure (*r* = 0.25, *p* < 0.01);
Trapp et al. [67] Aim: To examine whether personal strengths (optimism, sense of coherence, resilience) were associated with mental and physical health related quality of life in dementia caregivers	*N* = 130 dementia caregivers (*n* = 20 in Mexico *n* = 110 in Argentina). 77.7% were female, 76.9% were married. The mean age was 56.84 years (*SD* = 13.18) Instituto de Neurociencias de San Lucas, Argentina Baja California, Mexico (Not reported)	Cross-sectional design (Telephone interviews in Mexico, interviews in Argentina)	Personal strengths will be positively associated with both mental and physical health related HRQOL No hypotheses regarding expected differences in resilience between groups	Reliability not reported in this study sample A small correlation between resilience and mental health (*r* = 0.24, *p* < 0.01); medium correlations between resilience and sense of coherence (*r* = 0.41, *p* < 0.001); optimism (r = 0.48, *p* < 0.001) but not physical health. Linear regression of the personal strength measures found resilience predicted mental health (β = -0.25, *p* < 0.05), but the negative direction of the association is in contrast to the authors hypothesis. The results partially confirm the hypotheses
Trujillo et al. [68] (To use structural equation modelling (SEM) to investigate the role of family dynamics and personal strengths in the mental health of dementia caregivers from Latin America)	*N* = 110 carers; *n* = 83 female, mean age = 57.20 (SD = 13.47). Main relationship to person with dementia – spouse (*n* = 54), children (*n* = 50). *N* = 87 married Rosario, Argentina (Spanish)	Cross sectional design (Face to face interview)	It is hypothesized that personal strengths will mediate the relationship between family dynamics and caregiver mental health in a sample of dementia caregivers from Argentina	Ideal internal consistency (α = 0.97) Large correlation between resilience and satisfaction with life (*r* = 0.52, *p* < 0.01) Medium correlations between resilience and empathy (*r* = 0.37, *p* < 0.01), pathology (*r* = -0.41, *p* < 0.01), cohesion (*r* = 0.31, *p* < 0.01), communication (*r* = 0.35, *p* < 0.01), family satisfaction (*r* = 0.35, *p* < 0.01), sense of coherence (*r* = 0.44, *p* < 0.01), optimism (*r* = 0.49, *p* < 0.01), depression (r = -0.42, *p* < 0.01), anxiety (*r* = -0.40, *p* < 0.01) Small correlation between resilience and flexibility (*r* = 0.18, *p* < 0.01) The latent variable personal strengths, which included resilience, optimism and coherence was associated with mental health (β1.48, *p* < 0.001). Bootstrapping analyses with 2,000 calculated samples found that the indirect effect of family dynamics on caregiver mental health through personal strengths was also statistically significant (β = .99, *p* < .001)
**Brief Resilient Coping Scale (BRCS)**				
Jones et al. [69] Aim: To describe the demographic and psychosocial characteristics of caregivers, and which of these aspects may influence dementia café attendance)	*N* = 80 family caregivers of people living with a dementia. n = 21 male, *n* = 59 female. *N* = 52 were spouses, *n* = 28 ‘other’. Age range 30–80 + (no mean age reported) Type of dementia: *N* = 26 Alzheimer’s Disease; *n* = 12 vascular; *n* = 20 mixed (AD and vascular); *n* = 4 DLB/FTD; *n* = 6 ‘other’ Norfolk, England (English)	Cross-sectional between group design ( Self-completion)	Cafe attendees will ´ have greater wellbeing, resilience, and social support than non-attendees	Reliability not reported in this study sample Caregivers attending a dementia café reported higher resilience than non-attendees as hypothesised (mean difference –3.54, 95% CI –5.34 to 1.73; *p* < 0.001)
Jones et al. [70] (To compare socio-demographic characteristics and the availability of social support for carers with low and high resilient coping, and identify if any domain of social support predicted high resilient coping in informal carers of people with dementia)	*N* = 108 carers. The majority (69%) were women (69%). 61% of carers were aged 70 years or above (mean age not available for total sample). Spousal relationship was most common (61%) as was carer co-residence with the person with dementia (78%) Norfolk, England (English)	Cross-sectional design/postal survey (Self-completion)	1. Carers who report high resilient coping would have greater perceived social support 2. High resilient coping would be associated with emotional/informational support and tangible support in line with qualitative studies	Reliability not reported in this study sample Low resilient carers reported significantly less availability of emotional/informational support than high resilient carers (Mean rank difference = 20.17, U = 913.00, z = 3.35, *p* = 0.001) although the effect size was small (h = 0.10). The perceived availability of tangible support was lower for carers who report low resilient coping (Mean rank difference 14.77, U = 1059.00, z = 2.47, *p* = 0.014) with a small effect size (h = 0.06). Low resilient coping carers perceived they had less availability of positive social interaction than carers who had high resilient coping scores (Mean rank difference = 18.89, U = 947.5, z = 3.175, *p* = 0.001) and the effect was small (h = 0.09) Emotional/informational support had greatest influence on high resilient coping (OR = 1.92, 95%CI = 1.29 to 2.88, *p* = 0.001). Carers with greater access to tangible support were also more likely to be highly resilient copers (OR = 1.43, 95%CI = 1.07 to 1.91, *p* = 0.017). greater availability of affectionate support (OR = 1.49, 95%CI 1.10 to 2.00, *p* = 0.010) and positive social interaction (OR = 1.76, 95%CI = 1.24 to 2.49, *p* = 0.002) predicted high resilient coping. Gender was a significant predictor, with females being more likely to be high resilient copers (OR = 3.45, 95%CI = 0.448.27, *p* = 0.01) (The authors categorised high and low resilience groups through a mean score split, which is not the intention of the measure)
Meléndez et al. [71] (To ascertain whether patients with MCI and Alzheimer’s Disease experience changes in psychological wellbeing, resilience and coping compared to older adults without cognitive impairment	*N* = 32 healthy elderly people mean age = 73.9 years, *SD* = 5.05, range 65–87 years old, 31 amnestic mild cognitively impaired (aMCI) patients mean age = 75.93 years, *SD* = 6.23, range 64–88 years old, and 32 Alzheimer's disease (AD) diagnosed patients mean age = 76.84 years, SD = 4.57, range 65–83 years old Valencia, Spain (Spanish)	Cross-sectional between group design (Face to face interview)	None specified	Adequate to ideal internal consistency (α = 0.78) Significant differences in resilience (F(2,94) = 12.67; *p* = 0.001) between people living with Alzheimer’s Disease (M = 13.19), mild cognitive impairment (M = 15.69) and older people with no impaired cognition (M = 17.53). It is unclear as to why these differences might be expected
**Caregiver Resilience Scale (CRS)**				
Maneewat et al. (2016) [72] (1) To develop the caregiver resilience scale for Thai caregivers of older people with dementia. (2) To examine the validity and reliability of the caregiver resilience scale	Pre-test *N* = 30 carers; field test *N* = 150 carers No demographic data is presented. The inclusion criteria suggests they were between age 20–60 and able to speak Thai Upper Southern Thailand (Thai)	Study design unclear (two cross-sectional studies?) Mode of data collection unclear, semi-structured interview is suggested	None stated	Pre-test: The first draft of the CRS was composed of 36 items within six domains of (physical competence, relationship competence, emotional competence, cognitive competence, moral competence, and spiritual competence). This was derived from a scoping review and “semi-structured interview among 10 caregivers in order to confirm the pre-specified structure of caregivers’ resilience and congruence with Thai caregivers’ context” (the data is not presented for either of these) The draft was examined by three experts who recommended the deletion of six items, which were considered redundant (decision not explained). The authors developed a content validity form for the experts who rated each item (− 1 = not relevant, 0 = somewhat relevant, and 1 = quite relevant). They state that for the 30 items, the content validity index was 0.84, although the range and interpretation is not provided. Internal consistency from the 30 carers α = 0.87 Item and item, item and subscale, and item and total correlations were between 0.56 to 0.88 (data not presented) Field test: Internal consistency of the CRS was 0.87. The internal consistency of the subscales of the physical competence domain, relationship competence domain, emotional competence domain, moral competence domain, cognitive competence domain, and spiritual competence domain ranged from 0.52 to 0.87 (data not presented). The correlations of items and their subscale ranged from 0.32 to 0.83 (data not presented). The results of Bartlett’s test of sphericity showed a significant high inter—item correlation (χ2 = 17,124.13, *p* < 0.01). The communalities of the 30 items of factor extraction ranged from 0.40 to 0.78. The six components with initial eigenvalues greater than 1 ranged from 1.43 to 21.34, and the total variances at 63.67 is reported. (Some evidence of structural validity?) The rationale for the caregiver resilience scale is theoretically under-developed. The authors note there are only two measures for assessing resilience, stating that one (the CD-RISC) has only limited evidence in the general population (which is incorrect, as it is widely used) and another the Responses to Stressful Experiences Scale, has been mainly applied in the military. There are other measures of resilience (as identified in this review) They correctly note most of the resilience measures have been developed with Western population, and there is a gap for a resilience measure developed with and for Thai caregivers. The authors note the domains of the measure were identified through a concept analysis, although this work is not presented or the article cited. They state qualitative interviews were undertaken with 10 carers to ‘confirm’ the domains suggested by the concept analysis, however this data is not presented
Pandya et al. [66]	185 carers at the pre-test phase (96 in the intervention group and 89 in the control group) and 145 participants at the post-test phase (78 intervention group and 67 control group) Mean age pre test control group 52.5(10.67) intervention group 52.68(11.03), post test control group 57.6(11.32) intervention group 58.02(10.38) Females pre test control group *N* = 77/86.52%; pre-test intervention group N = 78/81.25%; post test control group *N* = 59/88.06%; *N* = 67/85.90% Male 12 13.48 18 18.75 8 11.94 11 14.10 Female 77 86.52 78 81.25 59 88.06 67 85.90 Mumbai, India and Kathmandu, Nepal (English)	Intervention study with post-intervention data collected five years later (Mode of data collection unclear)	Hypothesis 1: Meditation program would reduce the perceived caregiving burden overload and enhance self-efficacy and resilience of home-based caregivers of older adults with Alzheimer’s. Hypothesis 2: It is hypothesized that the intervention group participants’ characteristics as well as program-related characteristics would predict different levels of self-efficacy and burden. Hypothesis 3: Home practice by caregivers would be the strongest predictor of perceived caregiving burden reduction, caregiving self-efficacy enhancement and increased resilience	Cronbach’s α = .87; item-scale inter-correlation = .82 Post-test CRS scores of the intervention group were higher than the control group (mean difference = 27.68, *p* = .001, d = 4.54) and their own pre-test scores (mean difference = 27.5, *p* = .001, d = 4.65)

Fifty-one studies with 53 entries in Table 2 as two studies [42, 66] each use 2 measurement scales

## Description of the resilience measures

### Resilience scale (RS)

The RS [77] aims to measure the degree of individual resilience (considered a positive personality characteristic) that enhances individual adaptation. The target group is adults. The scale has two dimensions (personal competence and acceptance of self and life) measured by twenty-five positively worded items, each scored on a 7-point scale ranging from 1 (strongly disagree) to 7 (strongly agree). The total scores range from 25 to 175 with higher scores indicating higher resilience. Specifically scores greater than 145 indicate moderately high-to-high resilience, scores from 116 to 144 indicate moderately low- to-moderate levels of resilience, and scores of 115 and below indicate very low resilience [78]. Further clarification regarding the interpretation of scores, and suggestions for (self) improvement are provided by the authors and correspond with the following scoring ranges; 25–100 = very low, 101–115 = low, 116–130 = on the low end, 131–145 = moderate, 146–160 = moderately high, 161–175 = high [49]. The items were developed from qualitative research with 24 older women who successfully overcame a major life event. The target group were not involved in the item selection. The Resilience Scale is written at the 6^th^ grade level (12–13 years) and it is suggested completion takes about 5–7 min by most people. The scale has been widely applied across different age and patient groups, translated into other languages and demonstrated reliability and validity [78], and has a dedicated website where users can register and receive a copy of the measure (for a fee), plus detailed information regarding its development and application, how to score and interpret it. Cosco et al. [14] included two studies using this measure in older populations, reporting high internal consistency (α = 0.85 and α = 0.91). Windle et al. [13] scored this scale 6/18 in their quality assessment (the measure lacked data in relation to test–retest at that time). Both authors suggest the RS as a suitable measure for older adults, with the RS suggested by Cosco et al. as having the strongest evidence in older populations. Twelve studies in this review used the RS with caregivers (see Table 2).

### Brief resilience scale (BRS)

The BRS [79] aims to measure the ability to bounce back or recover from stress. The target group is adults. The scale has six items, scored on 5-point scale (total score range 6–30). The scale can be interpreted as low resilience (1–2.99), normal resilience (3–4.30), high resilience (4.31–5). The items were developed by the authors and refined through feedback from undergraduate students. Some of the target group (students) were involved in the item selection. Windle et al. [13] scored this scale 7/18 in their quality assessment. As the measure had evidence of test–retest, Windle et al. suggest the BRS could be useful for assessing change in response to an intervention. Ten studies in this review used the BRS with caregivers and one study [45] used the BRS with dyads (people with dementia and carers) (See Table 2). The scale is freely available for use and users should correctly cite and acknowledge the authors. The items and scoring are presented in the development papers.

### Resilience scale-14 (RS-14)

The RS-14 is a shortened version of the original RS. The 14-items derived from the RS are scored on a 7-point scale ranging from 1 (strongly disagree) to 7 (strongly agree) the same as the original RS. The total score ranges from 14 to 98 with higher scores indicating higher resilience. Specifically, scores greater than 90 in the RS-14 indicate high resilience, scores from 65 to 81 indicate moderately-low to moderate resilience, and scores of 64 and below indicate low resilience [78]. Further narrative interpretation and suggestions for (self) improvement are provided and correspond with the following scoring ranges; 14–56 = very low, 57–64 = low, 65–73 = on the low end, 74–81 = moderate, 82–90 = moderately high, 91–98 = high. The RS-14 is not comprised of any sub-scales. The original Resilience Scale and the RS-14 are strongly correlated (*r* = 0.97, *p* > 0.001 [78] and the measure is included in a dedicated website with the RS, where users can register and receive a copy of the measure (for a fee), plus detailed information regarding its development and application, how to score and interpret it. As a more recent development, no published studies using the RS-14 were identified in the reviews of Windle et al. [13] or Cosco et al. [13]. Five studies in this review used the RS-14 with caregivers and two studies used it with people living with dementia (see Table 2).

## Connor-Davidson resilience scale (CD-RISC)

The CD-RISC [80] aims to provide a self-rated assessment of resilience and a clinical measure to assess treatment response. The target group is adults. The scale has twenty-five items with no sub-scales, scored on a 5-point scale (total score range 0–100). The items were developed by the authors, derived from themes identified in a literature review, and the target group were not involved in the item selection. The scale has been widely applied across different age and patient groups, and translated into other languages, with a dedicated website about the development of the measure. Potential users are first required to register. The CD-RISC scored 7/18 in the review of Windle et al. [13]and was the only measure with data regarding responsiveness. Cosco et al. [14] concluded the CD-RISC potentially demonstrates sufficiently acceptable psychometric properties in older populations, although more psychometric evaluation studies are required. Seven studies in this review used the CD-RISC with caregivers (Table 2).

## Connor-Davidson resilience scale (CD-RISC 10)

The CD-RISC 10 [81] is a shortened version of the CD-RISC, being a single factor measure derived from a factor analysis of the full scale. The 10-items are scored on a 5-point scale ranging from 0 (*not true at all*) to 4 (*true nearly all the time*). The target group are adults and the measure was derived from data provided by 1,743 undergraduates from San Diego State University (SDSU) who completed questionnaires for course credit in 2004–2005. Most of these were female (74.4%) and the mean age was 18.8 years (*SD* = 2.2).

Cosco et al. [14] concluded the CD-RISC potentially demonstrates sufficiently acceptable psychometric properties in older populations, although more psychometric evaluation studies are required. Three studies in this review used the CD-RISC 10 with caregivers (Table 2).

## Dispositional resilience scale (DRS)

The DRS [82] aims to measure psychological hardiness and is an adaptation of an earlier measure of personality hardiness. The target group is adults. The scale has three sub-scales (commitment, control and challenge), with the full scale measured by 45-items, scored on a 4-point scale (total score range 0–135). No information is provided as to whether the target group were involved in the item selection. The DRS scored 4/18 in the review of Windle et al. [13]. One study in this review used the DRS with caregivers. The scale is not widely used, but appears to be in the public domain.

## Resilience scale for adults (RSA)

The RSA [83] aims to measure the protective resources that promote adult resilience and facilitate psychosocial adaptation to adversities. The scale has five dimensions (personal competence, social competence, family coherence, social support and personal structure) measured by 37 items, scored on a 5-point scale. Subsequent investigation reduced the questionnaire to 33 items scored on a 7-point scale, ranging from 33–231, and suggest the factor ‘personal competence’ might contain two factors ‘perception of self’ and ‘planned future’ [84]. It is unclear if the target population were involved in the item selection. The RSA scored 7/18 in the review by Windle et al. [13], with the measure demonstrating evidence of test–retest stability. Eight studies in this review used the RSA with caregivers. Users are advised to contact the authors for permission to use the scale, and it has been translated into a number of languages.

## Brief resilient coping scale (BRCS)

The BRCS [85] aims to measure resilient adaptive-coping behaviours in adults dealing with current stressors. The target group in scale development was people with Rheumatoid Arthritis. The scale has four items, scored on a 5-point scale (range 4–20). These can be interpreted as low resilient copers (4–13 score), medium resilient copers (14–16 score) and high resilient copers (17–20). The items were initially developed by the author and refined through consultation with six student nurses, the target group were not involved in the item selection. Cosco et al. [14] found one study examining the psychometric properties of the BRCS in an older Spanish population. They report the scale has good reliability and confirmatory factor analysis supported a one-factor structure, but the authors suggest further psychometric evaluation is required across other criteria. The scale is freely available for use and users should correctly cite and acknowledge the authors. The items and scoring are presented in the development papers. Three studies in this review use the BRCS, two with caregivers, and another comparing healthy older adults, adults with MCI and Alzheimer’s Disease (see Table 2).

## Caregiver resilience scale (CRS—identified in the review process)

The caregiver resilience scale was developed for Thai caregivers of older people living with dementia [72]. The scale has six dimensions of competence (physical, relationship, emotional, moral, cognitive, spiritual), measured by 30 items scored on a 4-point scale (total score range 0–30). The authors note the domains of the measure were identified through a concept analysis, although this work is not presented or the article cited in their paper. They state qualitative interviews were undertaken with 10 carers to ‘confirm’ the domains suggested by the concept analysis, but the analysis and results are not presented and they do not appear to be reported in another article. Further information is presented in Tables 2 and 3.

**Table 3:** The evaluative assessment scores for each study

	**Conceptual model**			**Content validity**			**Reliability**		**Construct validity**				**Scoring & interpretation**			**Respondent burden & presentation**		
**Author(s) (study population)**	**1**	**2**	**3**	**4**	**5**	**6**	**7**	**8**	**9**	**10**	**11**	**12**	**13**	**14**	**15**	**16**	**17**	**18**
	**Definition**	**Population**	**Expectations of structure**	**Target population involved**	**Experts**	**Item development**	**Internal consistency**	**Meet criteria**	**Single or multiple scale**	**Hypothesised associations**	**Hypothesised differences**	**Change over time**	**Scoring**	**Missing data**	**Interpretation**	**Time to complete**	**Literacy level**	**Measure available**
**Resilience Scale**																		
Bull [26] (Carers)	1	0.5	0	0	0	0	0	0	0	0	0	0	0	0	1	1	0	0
Dias et al. [27] (Carers)	1	0.5	0	0	0	0	0	0	0	0	0	0	0	0	1	0	0	0
Garity [73] (Carers)	1	0.5	0	0	0	0	0	0	0	0	0	0	0	0	1	0	0	0
Fitzpatrick & Vacha-Haase [28] (Carers)	1	1	0	0	0	0	1	1	0	1	0	0	0	0	1	0	0	0
Kimura et al. [29] (Carers)	1	0.5	0	0	0	0	1	1	0	0.5	0	0	0	0	1	0	0	0
Kimura et al. [30] (Carers)	1	0.5	0	0	0	0	0	0	0	0	0	0	0	0	1	0	0	0
MacCourt, et al. [31] (Carers)	1	0.5	1	0	0	0	1	1	0	0	1	1	0	0	1	0	0	0
Monteiro et al. [32] (Carers)	1	1	0	0	0	0	1	1	1	0	0	0	0	0	1	0	0	0
Pessotti et al. [33] (Carers)	1	0.5	0	0	0	0	0	0	0	0.5	0	0	0	0	0	0	0	0
Rosa et al. [34] (Carers)	1	0.5	0	0	0	0	0	0	0	0.5	0	0	0	0	1	0	0	0
Scott [74] (Carers)	1	0.5	0	0	0	0	0	0	0	0.5	0	0	0	0	1	0	0	0
Svanberg et al. [35] (Carers)	1	1	0	0	0	0	0	0	0	0	0	0	0	0	1	0	0	0
**Brief Resilience Scale**																		
Canevelli et al. [36] (Carers)	1	0.5	0	0	0	0	1	1	0	0.5	0	0	1	0	1	0	0	0
Chan et al. [38] (Carers)	1	0.5	0	0	0	0	0	0	0	0	0	0	0.5	0	1	0	0	0
Kalaitzaki et al. [39] (Carers)	1	0.5	0	0	0	0	1	1	0	0.5	0	0	1	0	1	0	0	0
McManus et al. [40] (Carers)	0	0.5	0	0	0	0	1	1	0	0	0	0	1	1	0	0	0	0
Prins et al. [41] (Carers)	1	0.5	0	0	0	0	0	0	0	0	0	0	1	0	1	1	0	0
Vatter and Leroi [45] (Dyad)	1	0.5	0	0	0	0	0	0	0	0.5	0	0	0	1	1	0	0	0
Vatter et al. [43] (Carers)	1	0.5	0	0	0	0	0	0	0	0.5	0	0	0	0	1	0	0	0
Vatter et al. [44] (Carers)	1	1	0	0	0	0	0	0	0	0	0	0	0	1	1	0	0	0
Wuttke-Linnemann et al. [46] (Carers)	1	0.5	1	0	0	0	0	0	1	0.5	1	0	1	0	0	0	0	0
Sutter et al. [42] (Carers)	1	0.5	0	0	0	0	0	0	0	0.5	0	0	1	0	1	0	0	0
**RS-14**																		
D’Onofrio et al. [47] (People with dementia)	1	0.5	0	0	0	0	0	0	0	0	0	0	0	0	0	0	0	0
McGee et al. [48] (People with dementia)	1	1	0	1	1	1	1	1	0	0.5	0	0	0	0	0	0	0	1
Orgeta et al. [49] (Carers)	0	0.5	0	0	0	0	0	0	0	0	0	0	0	1	1	0	0	0
Sánchez-Teruel et al. [50] (Carers)	1	0.5	1	0	0	0	1	1	0	0	0	0	0	1	0	0	0	0
Stansfeld et al. [51] (Carers)	0	0.5	0	0	0	0	0	0	0	1	0	0	0.5	0	1	0	0	0
Wilks et al. [52] (Carers)	1	0.5	0	1	1	0	0	0	0	1	0.5	0	0.5	1	1	0	0	0
Wilks et al. [75] (Carers)	1	0.5	0	0	0	0	1	1	0	0	0	0	0	1	1	0	0	0
**CD-RISC**																		
Duran-Gomez et al. [53] (Carers)	1	0.5	0	0	0	0	0	0	0	0.5	0	0	0	0	1	0	0	0
Gómez-Trinidad et al. [54] (Carers)	1	1	0	0	0	0	0	0	0	0	0	0	0	1	1	0	0	0
Lavretsky et al. [55] (Carers)	0	1	1	0	0	0	0	0	0	0	0	0	0	0	1	0	0	0
Rivera-Navarro et al. [56] (Carers)	0	0.5	0	0	0	0	0	0	0	1	0	0	0	0	0	0	0	0
Ruisoto et al. [57] (Carers)	0	0.5	0	0	0	0	0	0	0	0	0	0	0	0	1	0	0	0
Serra et al. [58] (Carers)	1	0.5	0	0	0	0	0	0	0	0.5	0	0	0	0	0	0	0	0
Wilks and Vonk [76] (Carers)	1	0.5	0	0	0	0	0	0	0	0	0	0	0	0	1	0	0	0
**CD RISC-10**																		
Bravo-Benitez et al. [59] (Carers)	0	0.5	0	0	0	0	0	0	0	0	0	0	0	0	0	0	0	0
Carbone et al. [37] Carers	1	0.5	0	0	0	0	0	0	0	0.5	0	0	1	0	1	0	0	0
Sarabia-Cobo & Sarria [60] (Carers)	0	1	1	0	0	0	1	1	0	0.5	0	0	0.5	0	1	0	0	0
**DRS**																		
O’Rourke et al. [61] (Carers)	1	1	1	0	0	0	0	0	1	0.5	0	0	0	0	0	0	0	0
**RSA**																		
Altieri & Santangelo [62] (Carers)	1	0.5	1	0	0	0	0	0	0	0.5	0	0	0	0	1	0	0	0
Elnasseh et al. [63] (Carers)	1	0.5	1	0	0	0	1	1	0	0.5	0	0	0	0	1	0	0	0
Gulin et al. [64] (Carers)	1	0.5	1	0	0	0	1	1	0	0.5	0	0	1	0	1	0	0	0
Pandya [66] (Carers)	0	1	0	0	0	0	1	1	0	0	0.5	1	0	0	1	0	0	0
Senturk et al. [65] (Carers)	1	0.5	1	0	0	0	1	1	0	1	0	0	0	0	1	0	0	0
Sutter et al. [42] (Carers)	0	0.5	1	0	0	0	0	0	0	0.5	0	0	0.5	0	1	0	0	0
Trapp et al. [67] (Carers)	1	0.5	1	0	0	0	0	0	0	0.5	0	0	0	0	0	0	0	0
Trujillo et al. [68] (Carers)	1	1	1	0	1	0	1	1	0	1	0	0	0	0	0	0	0	0
**BRCS**																		
Jones et al. [69]	1	0.5	0	0	0	0	0	0	0	0	1	0	0	0	1	0	0	0
Jones et al. [70]	1	0.5	0	0	0	0	0	0	0	1	0	0	0	0	1	0	0	0
Meléndez et al. [71] Older people/MCI/AD	1	1	0	0	0	0	1	1	0	0	0	0	0	0	0	0	0	0
**Caregiver Resilience Scale**																		
Maneewat et al. [72] (carers)	1	1	0	1	1	0	1	1	1	0	0	0	0	0	1	0	0	1
Pandya [66] (Carers)	0	1	0	0	0	0	1	1	0	0	0.5	1	0	0	1	0	0	0

• Scores represent the adapted scoring criteria outlined in Table 1

• Francis et al. (2016) recommends a total score is not derived, as the scoring criteria items are not necessarily equally weighted

## Psychometric assessment

To facilitate evaluation of the nine resilience measures, Additional File 2 synthesises the psychometric assessment for each study into a narrative summary for each resilience measure across the six domains of the scoring criteria, which are discussed below The individual scores for each studyfollowing assessment against the six domains of the scoring criteria are presented in Table 3.

## Conceptual model

Most of the studies defined the target population of their studies and briefly defined the construct to be measured (resilience), with a wide range of definitions used but did not expand on the theoretical basis of resilience. With the exception of the eight studies using the RSA, the extent to which resilience was conceptualised as a single construct/scale or a multiple construct/subscales was not addressed. This is a particular issue for further attention in the CRS [72], which has been developed for dementia carers. The conceptual model is especially important in the development of measures, where the theoretical underpinnings should be hypothesised. There is a growing literature suggesting what might constitute resilience in dementia caregivers (e.g. [9–11]) who indicate that social, psychological/individual and structural aspects are important. This then raises the question as to whether existing measures of resilience adequately reflect the theoretical underpinnings in this population, especially as Windle et al. [13] found that most resilience measures focus mainly on the individual/psychological aspects of resilience. Exploratory work with people living with dementia suggests the conceptualisation of resilience as ‘bouncing back’ from adversity, reflected in some resilience measures, may not be appropriate when living with a degenerative condition [86]. Other exploratory research notes that ‘growth’ was mentioned by several dementia carers when explaining why they considered themselves resilient, however there is debate in the literature regarding whether definitions of resilience should include concepts such as ‘growth’ or ‘thriving’ [87]. Further theoretical work with both populations exploring what resilience means to them would usefully contribute to the developing conceptual basis of resilience, and the extent to which existing measures are conceptually appropriate.

## Content validity

The domain ‘content validity’ builds on the conceptual model to address the extent to which the questions and any sub-scales reflect the perspectives of the target group, and should involve the target group and content experts in their development [21]. Most of the studies scored poorly on this domain as they had applied an existing resilience measure developed in a different population in their studies, and the assessment items are primarily designed for measurement development. In terms of the original development of the measures identified in this review, only the RS involved the target group (older people) in its original development, which would also extend to the shorter RS-14.

Two studies in this review, one with carers [52] and another with people living with dementia [48] attended to the content validity of the RS-14, where adaptations are reported in response to the pilot work with the new target groups. The adaptations reported by McGee et al. [48] are potentially helpful for difficulties with comprehension and communication. Another reported how they used forward-back translation to translate the RSA into Spanish and obtained further feedback for any additional modifications by researchers familiar with the regional language and culture in Argentina [68].

Adapting existing measures is potentially efficient and pragmatic but requires careful consideration. The conceptual model of a measure for the target population should be relevant. Simplifying questions and response scales could potentially mean the measure is now a different tool, no longer comparable with the original. Some measures are subject to copyright, and the developers may not support adaptations. Researchers are encouraged to engage with the developers of the original measures from the outset should they wish to undertake adaptations.

The CRS provided evidence of content validity for two of the three assessment items, indicating 10 caregivers were asked to confirm the pre-specified structure of caregivers’ resilience as relevant to Thai caregivers, and three experts were consulted on the content validity [72]. No data is presented to support this process nor any information about how the items were initially generated. The conceptual basis of this measure requires further explanation. Given the theoretical issues described in the previous section, the extent to which the resilience measures are relevant and comprehensively represent resilience in these two populations requires further investigation.

## Reliability

The internal consistency of the measures was assessed under the domain ‘reliability’. Nineteen studies across seven measures provided this data from their study populations, with no data relating to internal consistency for the CD-RISC, and the DRS. Some studies cited the internal consistency of the original development study. Additional File 2 summarises how many studies report this data for each of the nine measures. Where reported, the available data note internal consistency in the ‘ideal’ and ‘adequate to ideal’ range for the measures in carers, in one study for the RS-14 in people with dementia [48] and in another for the BRCS across a mixed sample of healthy older adults, people with MCI and Alzheimer’s Disease [71]. One study [39] reported low internal consistency for the BRS in carers out of the three that provided the data for this measure. When measures are applied in a different population to the one previously developed such as those identified in this review, as a minimum we encourage authors to report the internal consistency of the measure. Reporting reliability scores of original measures are insufficient if using a measure with a new population.

## Construct validity

The conceptual aspect also has implications for the domain of ‘construct validity’, which reflects the extent to which a scale measures the construct of resilience. Most of the studies in this review provided some data for this domain, mainly around associations with other existing outcome measures or demographic data, and we used Cohen’s criteria [24] to indicate the size of effect and consequent strength of the relationship (Table 2). Twenty-eight studies hypothesised effects (associations with other outcome measures or demographic data, or differences in scores between relevant groups). These were partially supported. The others were either exploratory or did not specify hypotheses. The assessment of construct validity required authors to state hypotheses regarding expected correlations, differences and the magnitude of these *apriori*, consequently, it is unclear what some authors were expecting to find from their analyses. For example, McGee et al. [48] state they test the convergent validity of their positive psychology measures, and the discriminant validity between the positive psychology measures, depression and anxiety, but do not hypothesise whether they expect a positive or negative relationship, or no relationship (which would be expected for discriminant validity).

Nine studies had a longitudinal aspect to their study design. Four hypothesised change in response to an intervention, with some evidence for the RSA and CRS in carers [65], an RCT found no effects of an intervention on the RS-14 in carers [49], and another reported improvements over time in the CD-RISC for carers but the data is not presented to support this claim [54]. There was partial support from a longitudinal cohort study for the DRS in carers [61]. Three did not specify hypotheses, but report improvements in the RS for carers in a grief coaching intervention [31], improvements in the CD-RISC 10 for carers in a grief intervention [59] and no difference in the BRS for carers in an arts intervention [40]. Test–retest data is required for the measures in these populations to help evidence stability of the measures when no change is anticipated. This will help confirm any changes found in response to an intervention are not random but an effect of the intervention.

Some studies are also likely to have been insufficiently powered to detect small effects which could provide support for construct validity. For example, with a power of 0.80 (β = 0.20) and α of 0.05 a sample of 85 participants is needed to detect a small-medium sized correlation of r = 0.3 [88]. To illustrate, Kimura et al. [29] did not find hypothesised associations between carers resilience and clinical characteristics of the care recipient but reported significant correlations between carer resilience and carer indicators of mental health. All correlations with resilience reported in this study, with the exception of the carer’s depression score (*r* = -0.405), had effect sizes below 0.35 which the study sample size (*n* = 43) was too small to detect using standard α = 0.05, β = 0.20 parameters. The insufficient powering of this study therefore reduces the likelihood that the statistically significant results showing associations between resilience and anxiety, and resilience and hopelessness reflect true effects, and may also mean that failure to find significant hypothesised relationships between resilience and other measures could be false negatives reflecting small sample size. Studies with small sample sizes therefore need to be interpreted with caution, and study sample size should be considered when weighing evidence in the evaluation of measure validity. Future research should aim to clarify how resilience is expected to influence outcomes through establishing hypotheses, derived from a sound theoretical basis, and should ensure that studies are sufficiently powered to detect expected effects.

## Scoring and interpretation

Most of the studies lacked information on how the total score for the measure used was derived (although this should be available in the original measure development papers) and how missing data was dealt with. There were some inconsistencies between the original development papers and the studies in this review in descriptions of cut-points. Kimura et al. [30] describe cut points of the RS measure in their study that indicate low, medium and high resilience, but these do not correspond with the suggested cut points of the original measure. The mean score (5.50) for the RS reported by Fitzpatrick and Vacha-Hasse [28] does not appear to reflect the scoring range as proposed by the developers, and it is unclear how this score was derived. Vatter et al. report data according to low (1–2.99) and high (3–5) BRS scores, but these categories are different to those specified by the scale developers who note low resilience (1–2.99), normal resilience (3–4.30), high resilience (4.31–5). There was also some lack of clarity regarding whether the original measure had been correctly administered, e.g. Sutter et al. [42] indicate using a 36-item version of the RSA, but further on state they removed seven items from a larger, 45-item scale. Lack of detail regarding changes to the scoring also featured. Stansfield [51] note the scoring of the RS-14 items as ranging between 1 (Strongly disagree) and 6 (strongly agree), with possible sores ranging from 14 to 84. This is different to the original measure, but no adaptations are described. McGee [48] note they adapted the RS-14 to a 3-point Likert scale (disagree, neither agree nor disagree, agree) from the original 7-point scale, but no information is provided regarding the scale range and how it was scored.

Future studies should ensure they are using the measure as recommended by the developers, and fully report any adaptations they make to aid interpretation and for future use by others.

## Respondent burden and presentation

This domain had limited information on time to complete the measure, with only one study, Bull [26], indicating the RS took 5–10 min to complete. None alluded to the literacy level of the original measure. Although these items are likely of more relevance to developers of new measures, when applied in new populations who may have different education and literacy levels than the populations the measure was developed for, this information would be useful to record, as any difficulties could invalidate the measure. One study provided the full measure in their paper [72]. The other measures are either available freely from the developers or for a fee.

## Implications of the results for research and practice

Identifying existing measures and examining the extent to which they are appropriate for use in a different population is a recommended first step in measurement development, to avoid the considerable time required for the development of a new measure [1]. This requires the validity and reliability to be established in the new population. Only one study explicitly set out to explore the validity of the RS-14, presenting some limited evidence of convergent validity without clear hypotheses [48]. Most of the papers presented a limited amount of relevant data for our assessment, highlighting areas to consider regarding the conceptualisation of resilience and the future application and development of resilience measures in these populations.

Based on this review, it is difficult to make firm recommendations regarding which measure may be most appropriate, and users should consider the context in which they wish to use it. We make some suggestions, recognising that people need to make pragmatic choices for research and practice regarding outcome measures. Where reported, the internal consistency was graded ‘adequate to ideal’ in all the measures except for one study [39]. Additional File 2 summarises how many studies report this data for each measure. Most of these are applied with carers, and the (limited) data suggests the RS, BRS, RS-14, RSA, CD-RISC 10 and CRS are reliably assessing the target construct in carers, and the RS-14 and the BRCS in people with dementia.

The evidence for construct validity was mixed, and for studies with hypotheses, there were mixed results regarding the effects, but these provide a useful starting point for validating in future studies. For example, there is some suggestion the resilience measures were associated with measures of depression, anxiety, burden and quality of life across the studies, with effects ranging from small to large.

For those wishing to measure the impact of services and interventions, evidence of responsiveness to the effects of an intervention was limited in this review as most of the studies were cross sectional. Of the studies that hypothesised change over time in response to an intervention, there was evidence from one study each for the RSA and the CRS.

This is an important area for further development, especially for researchers and practitioners who wish to measure changes in resilience in response to interventions and services.

As service evaluations often assess multiple outcomes, it may be prudent to consider efforts to minimise respondent burden by selecting a shorter resilience measure. The RS-14, a widely applied measure in general research and the short version of the RS was used in sixstudies with people living with dementia, providing some (albeit limited) evidence of construct validity. The BRS may also be a useful option with carers, but the focus on bouncing back raise questions regarding how appropriate this measure would be for people living with dementia. The RSA and the CD-RISC were viewed as the more psychometrically robust tools in the measures reviews of Cosco et al. [14] and Windle et al. [13], but evidence of their psychometric properties was lower in this present review. This may be due to the application of a different quality assessment criteria, but is more likely due to the included studies not reporting data relevant for psychometric assessment.Whatever the choice, this review calls for researchers and practitioners to report psychometric data to advance the field. For those with an interest in the field of positive psychology, the Positive Psychology Outcome Measure (PPOM) is validated for use in people living with dementia and may be especially useful for intervention studies [89].

A broader consideration is that dementia is a progressive disease affecting cognitive functioning, which presents challenges when developing and administering outcome measures. Elsewhere, researchers have demonstrated that people in the milder to moderate stages of the condition are capable of providing reliable responses on widely used outcome measures [e.g. [90, 91] and should be given the opportunity to provide their opinions. As with all degenerative conditions, there will be a point when psychometric assessment may not be possible. At this point, proxy measures which enable another person to provide responses on behalf of a person living with dementia are one option to overcome this, yet none of the resilience measures in the review have a proxy version available. The extent to which people with dementia may be unaware of difficulties or changes they are experiencing may influence reporting outcomes. Other research suggests that people with dementia who focussed less on memory problems, perhaps appearing less aware of difficulties, also reported better well-being and mood [92]. Although this effect may be interpreted as a form of positive response bias, it may also be viewed as an adaptive form of coping in some situations, focusing on strengths rather than problems [93].

Researchers and practitioners may also need to consider the specific type of dementia a person is living with, and whether simple adaptations in the administration of a measure may be required to best support a person with different clinical presentations. Working with the target groups in any adaptations or development of new measures will help ensure questions are appropriate and understandable.

## Strengths and weaknesses of this review

Following piloting of the checklist [21], additional criteria were developed to assist in applying the checklist to studies that used an existing resilience measure, such as those identified in this review. Unfortunately, the scoring of the included studies was hindered by the absence of psychometric information in some of the studies, and in this respect, scores reflect how well publications report psychometric information. This also does not necessarily mean the resilience measure is not suitable, and we encourage future users of resilience measures in these populations to report information to advance knowledge and inform further reviews. Given the number of studies identified it was beyond the resources and the timescale of this work to contact individual authors for further information.

The checklist could also be considered overly stringent. For example, a checklist item under the ‘conceptual model’ domain requires studies to state whether a single scale or multiple sub-scales are expected. Whilst a number of studies noted the number of items in the measure they applied, they did not explicitly state whether it was a single-scale or had sub-scales. The domain of ‘respondent burden and presentation’ reflects the time taken to complete and the complexity of a measure, and the extent to which a measure is in the public domain. With the exception of one study using the RS which reported completion time, it was not possible to ascertain this information. We suggest this domain is more relevant to measurement development than the application to studies using a measure, as this information is not usually included in primary research, and when included often refers to total study time rather than individual measures. However, few checklists are available and the application of the checklist in this review enabled a systematic psychometric assessment of the resilience measures in these populations.

We did not include ‘cognitive impairment’ (nor any truncations/derivatives) in the search terms. This was due to the phrase capturing literature on cognitive impairment relating not only to dementia, but also other neurological conditions such as stroke and/or learning difficulties, which remain outside the scope of the current review. We note however that including Alzheimer’s Disease and other variations would enrich the breadth of papers and extended our clinical population search terms to incorporate Alzheimer’s Disease (including rarer variants such as Posterior Cortical Atrophy).

## Conclusions

This study systematically identified nine resilience measures applied in 51 studies examining the resilience of people living with dementia and their carers. We critically reviewed the measurement properties and applicability from the available psychometric data using a standardised checklist adapted for purpose. To our knowledge, no previous study has undertaken this research and our work contributes important new findings.

Notably most of the studies (*N* = 43) were cross-sectional designs and most studies used resilience measures with dementia carers (*N* = 47). All the identified measures require further psychometric evaluation in both these populations, and we encourage researchers to report relevant data in their publications to help advance the evidence base. With the exception of the CRS (which requires further psychometric evaluation) the measures were not developed with, and for dementia carers and would benefit from further investigation so as to support their use in future research and practice. This could ensure the existing measures comprehensively reflect their personal experiences of resilience, together with the growing conceptual understanding of resilience in this population.

Only three studies measured the resilience of people living with dementia, with one study measuring the resilience of both carers and the person with dementia. Further research to understand the experience of resilience for people living with dementia is warranted. This could establish the extent these experiences are reflected in current measures in terms of the underpinning conceptual model, whether existing measures could be adapted and updated and whether a proxy version could be developed. Further work to establish a new measure may need to consider measuring resilience beyond the individual and include their families and communities as sources of resilience, reflecting contemporary thinking in international policy which recognises that resilience can be strengthened at three levels: individual, community and system/society [94] as corroborated in a systematic review examining the conceptual basis of resilience [6]. Practitioners might be served to consider these broader conceptual aspects of resilience in assessing and formulating support for this population. People living with dementia and carers should be central to any measurement development or adaptation, in order to embed their lived experiences. 

## Supplementary Information

**Additional file 1.** 

**Additional file 2.** 

## Data Availability

This article was derived from secondary sources (published research articles) which are cited in the reference list. No primary data is included. All data generated or analysed during this study are included in this published article [and its supplementary information files].

## Footnotes

1 Early detection and timely INTERvention in Dementia.
