# Supplementary material for: A systematic review and psychometric evaluation of resilience measurement scales for people living with dementia and their carers
Source: BMC Med Res Methodol. 2022 Nov 19;22:298. doi: 10.1186/s12874-022-01747-x (PMC9675235; doi:10.1186/s12874-022-01747-x)
Supplement: Supplementary file 1 — Additional file 1. [file 12874_2022_1747_MOESM1_ESM.docx]

**Supplementary material**

**Additional File 1: Further explanation of the modifications to the checklist**

**Conceptual model**: The section on ‘intended respondent population’ was expanded to extract data on a) study population, b) original measure population, and c) measure suitability for the study population. A score of 1 was given if papers described both the study population, and the population the measure was originally developed with or discussed the suitability of the measure for use with the study population. A 0.5 score was given for papers which only discussed their study population, but not the original measure population, and scored 0 if they did not discuss either study or original populations.

**Content validity:** all three sections on measure content development were expanded to extract data in relation to a) the study, b) the original measure, and c) suitability of original involvement/methodology for study population. Papers scored 1 if they reported content development in relation to their study, 0.5 if only in relation to the original measure development, and 0 if they didn’t discuss content development at all.

**Reliability:** extracted data on reliability included a) data on the study population, b) the original measure, and c) suitability of existing reliability indices for study population. Papers scored 1 if they reported reliability in relation to their study and 0 if there is no discussion of reliability. Assessment of reliability adequacy was: ideal r>=0.80; adequate r>=0.70; or lower if justified.

**Construct validity**: the sections on expected correlations with existing PRO measures/clinical data and expected difference in scores between known groups were expanded to extract data on ai) known associations aii) a priori hypotheses, b) study results, c) matching of results with reported known associations or hypotheses. Papers scored 1 if they reported known associations and reported results which matched these associations, 0.5 if they reported a priori hypotheses and reported results which matched these hypotheses, or if only some of the results matched the known associations/hypothesis, and 0 if they didn’t report any known associations/ a priori hypotheses, or results, or none of the results matched the known associations/hypotheses. Although not proposed by Francis et al., we established additional evaluative indicators to indicate the strength of the relationship between two measures using Cohen’s criteria (1992) where large correlations are > 0.50, medium correlations range between 0.30-0.49 and small correlations range between 0.10-0.29.

**Scoring and interpretation**: was expanded to extract data on a) how study scored/interpreted, b) if measure scoring/interpretation is as originally designed. A criterion-met score of 1 was only given for each section if all sub-sections met criterion or if an explanation for adaptions was clearly given, a 0.5 score was given if information on the scoring and interpretation was given for the study, but wasn’t discussed in relation to the original measure. The section on missing responses was as per the original. Extracting data on original measures is important because not all studies use the measure as originally designed or intended, potentially invalidating the reliability and validity of the original measure, with clear discussion of adaptations not always given.

Cohen, J. (1992). A power primer. Psychological Bulletin, 112, 155-9.
