# Supplementary material for: A systematic review and psychometric evaluation of resilience measurement scales for people living with dementia and their carers
Source: BMC Med Res Methodol. 2022 Nov 19;22:298. doi: 10.1186/s12874-022-01747-x (PMC9675235; doi:10.1186/s12874-022-01747-x)
Supplement: Supplementary file 2 — Additional file 2. [file 12874_2022_1747_MOESM2_ESM.docx]

**Additional File 2: Narrative summary of the psychometric appraisal**

| **Conceptual**  **model** | **Resilience Scale (N=12)** | **BRS**  **(N=10)** | **RS-14**  **(N=7)** | **CD-RISC**  **(N=7)** | **CD-RISC**  **10 (N=3)** | **DRS**  **(N=1)** | **RSA**  **(N=8)** | **BRCS**  **(N=3)** | **CRS**  **(N=2)** |
| --- | --- | --- | --- | --- | --- | --- | --- | --- | --- |
|  | All included a short definition of resilience and outlined the target population for their studies. 9/12 defined their population but did not reference the original measures population. 1/12 discuss scale structure. | 9/10 included a short definition of resilience. All outline the target population for their studies. 1/10 discuss scale structure. | 5/7 included a short definition of resilience. All studies defined their target population. 1/7 discussed scale structure. | 4/7 included a short definition of resilience, all define their target population, 1/7 discuss scale structure. | 1/3 defined resilience. 2/3 studies  defined their target population. One discussed scale structure. | The authors define resilience and the target population, and specify the three sub-scales for their analysis. | 6/8 included a short definition of resilience, all defined their target population and describe the five sub-scales. | All studies defined resilience and their target population, and note the measure is a single scale. | Both studies defined their target population, 1 a short definition of resilience. Both lack information on whether a single construct / scale or multiple subscales are expected. |
| **Content**  **validity** | **Resilience Scale (N=12)** | **BRS**  **(N=10)** | **RS-14**  **(N=7)** | **CD-RISC**  **(N=7)** | **CD-RISC**  **10 (N=3)** | **DRS**  **(N=1)** | **RSA**  **(N=8)** | **BRCS**  **(N=3)** | **CRS**  **(N=2)** |
|  | None of the studies noted any discussions with carers about the relevance of the measure. Three studies use a Portuguese version of the RS, which appears to have been adapted for use with a population of students in public schools in Rio de Jeneiro (information on cultural adaptation not presented). | None of the studies noted any discussions with carers about the relevance of the measure. | McGee et al. (2017) piloted the measure with four people with early-stage dementia, and subsequently reduced the Likert scale to three responses from seven, provided a colour-coded stimulus book to support visual and verbal administration and simplified some of the language. Wilks et al., (2018) note the data collection instrument and plans were ‘initially formulated and critiqued by administrative staff, targeted caregivers from their constituency, and the study's researchers’ (p.664) but no amendments are reported. | None of the studies note any discussions with carers about the relevance of the measure. | None of the studies note any discussions with carers about the relevance of the measure. | There is no reference to discussions with carers about the relevance of the measure. | None of the studies report any discussions with carers about the relevance of the measure, or if involved in any cultural adaptations, although the adaptions indicate experts involved. Different versions of the RSA are used. Sutter et al, 2016 use the 33-item version, the other studies use a 36-item version. | None of the studies report any discussions with carers or people with dementia about the relevance of the measure. Melendez et al. (2018) refer to an earlier study which confirmed the factor structure of the BRCS in an older Spanish population. | One of the studies notes some involvement of carers and experts in the development. |
| **Reliability** | **Resilience Scale (N=12)** | **BRS**  **(N=10)** | **RS-14**  **(N=7)** | **CD-RISC**  **(N=7)** | **CD-RISC**  **10 (N=3)** | **DRS**  **(N=1)** | **RSA**  **(N=8)** | **BRCS**  **(N=3)** | **CRS**  **(N=2)** |
| **(See Table 2**  **for data)** | 4/12 studies report reliability data, which was in the ‘ideal’ range for three studies and adequate to ideal for one study. | 3/10 studies report reliability, with two in the ‘ideal’ range and another in the ‘low’ range. | 3/6 studies report reliability in the ‘ideal’ range. | No reliability data are reported. | 1/3 reports reliability in the adequate to ideal range. | No reliability data are reported. The authors cite reliability data from a study of widowed older women, the implication being the measure is reliable in a similar population. | 5/8 studies report reliability in the ideal range. | One reports adequate to ideal reliability data. | Both report ideal reliability. |
| **Construct**  **validity** | **Resilience Scale (N=12)** | **BRS**  **(N=10)** | **RS-14**  **(N=7)** | **CD-RISC**  **(N=7)** | **CD-RISC**  **10 (N=3)** | **DRS**  **(N=1)** | **RSA**  **(N=8)** | **BRCS**  **(N=3)** | **CRS**  **(N=2)** |
| **(See Table 2**  **for data)** | 6/12 studies hypothesised effects, others explore associations between resilience and other measures. 11/12 were cross-sectional;  1 factor analysis of sub-scales;  One provides some evidence of change over time but not hypothesised. | 4/10 studies hypothesised effects; others explore associations between resilience and other measures. 9/10 were cross-sectional.  Change over time not ascertained in the intervention study (and not hypothesised). | 3/6 studies hypothesised expected effects, 1 set out some expectations.  4/6 cross-sectional; 1 pre-post design showed change over time (not hypothesised), 1 RCT found no change as hypothesised. | 4/7 hypothesised effects, 6/7 cross-sectional; 1 small intervention study states resilience improved as per hypothesis, but no data is presented. | 2/3 hypothesised effects; 1 intervention study, with limited evidence of change (not hypothesised). | The study used the three sub-scales of resilience, and specified hypotheses with some supportive evidence, including change over time. | 5/8 hypothesised effects; 7/8 cross-sectional; 1 intervention study with evidence of change over time as hypothesised. | 2/3 hypothesised effects; 3/3 cross sectional so not able to provide evidence of change over time. | 1/2 hypothesised effects; 1 intervention study with some evidence of hypothesised change over time. |
| **Scoring and interpretation** | **Resilience Scale (N=12)** | **BRS**  **(N=10)** | **RS-14**  **(N=7)** | **CD-RISC**  **(N=7)** | **CD-RISC**  **10 (N=3)** | **DRS**  **(N=1)** | **RSA**  **(N=8)** | **BRCS**  **(N=3)** | **CRS**  **(N=2)** |
|  | 0/12 reported how they derived the measure or dealt with  missing responses. 11/12 reported how the scores should be interpreted. | 7/10 reported how they derived the measure; 3/10 reported how they dealt with missing responses. 7/10 reported how the scores should be interpreted. | 2/7 provide some description of how they derived the measure; 4/7 report how they dealt with missing data; 4/7 reported how the scores should be interpreted. | 0/7 reported how they derived the measure; 1/7 reported how they dealt with missing data; 5/7 reported how the scores should be interpreted. | 2/3 reported how they derived the measure and how the scores should be interpreted; 0/3 reported how they dealt with missing data. | They do not report how the total score was derived and how it should be interpreted. There is no mention of missing data. | 2/8 reported how they derived the measure; 0/8 reported how they dealt with missing data; 6/8 report how the scores should be interpreted. | 0/3 reported how they derived the measure or dealt with missing data; 2/3 report how the scores should be interpreted. | 0/2 reported how they derived the measure or dealt with missing data; both report how the scores should be interpreted. |
| **Respondent burden and presentation** | **Resilience Scale (N=12)** | **BRS**  **(N=10)** | **RS-14**  **(N=7)** | **CD-RISC**  **(N=7)** | **CD-RISC**  **10 (N=3)** | **DRS**  **(N=1)** | **RSA**  **(N=8)** | **BRCS**  **(N=3)** | **CRS**  **(N=2)** |
|  | 1/12 noted how long the measure took to complete. 0/12 noted the expected literacy level or if the measure was publicly available. | No information available in relation to this domain. | 0/7 noted how long the measure took to complete. 0/7 noted the expected literacy level. 1/7 note the adapted measure, stimulus book and instructions for administration are available via request (McGee et al.) | 0/7 noted how long the measure took to complete. 0/7 noted the expected literacy level. 0/7 did not note if the measure was publicly available. | No information available in relation to this domain. | No information available in relation to this domain. | No information available in relation to this domain. | Not possible to ascertain from these studies. | 0/2 noted how long the measure took to complete. 0/2 noted the expected literacy level. 1/2 present the measure in their paper. |

*Summaries derived from 53 psychometric assessments from 51 studies across nine measures (Table 3)
